# *Clostridioides difficile* canonical L,D-transpeptidases catalyze a novel type of peptidoglycan cross-links and are not required for beta-lactam resistance

**DOI:** 10.1016/j.jbc.2023.105529

**Published:** 2023-12-01

**Authors:** Nicola F. Galley, Darren Greetham, Marcel G. Alamán-Zárate, Mike P. Williamson, Caroline A. Evans, William D. Spittal, Jessica E. Buddle, Jane Freeman, Georgina L. Davis, Mark J. Dickman, Mark H. Wilcox, Andrew L. Lovering, Robert P. Fagan, Stéphane Mesnage

**Affiliations:** 1 School of Biosciences, University of Sheffield, Sheffield, UK; 2 Department of Chemical and Biological Engineering, University of Sheffield, Sheffield, UK; 3 Department of Microbiology, Leeds Teaching Hospitals NHS Trust, Leeds Institute of Medical Research, University of Leeds, Leeds, UK; 4 Healthcare Associated Infections Research Group, Leeds Institute of Medical Research University of Leeds, Leeds, UK; 5 School of Biosciences, University of Birmingham, Birmingham, UK

**Keywords:** *Clostridioides difficile*, peptidoglycan, L,D-transpeptidase, cross-link, bacterial cell envelope, antibiotics, beta-lactams

## Abstract

*Clostridioides difficile* is the leading cause of antibiotic-associated diarrhea worldwide with significant morbidity and mortality. This organism is naturally resistant to several beta-lactam antibiotics that inhibit the polymerization of peptidoglycan, an essential component of the bacteria cell envelope. Previous work has revealed that *C. difficile* peptidoglycan has an unusual composition. It mostly contains 3-3 cross-links, catalyzed by enzymes called L,D-transpeptidases (Ldts) that are poorly inhibited by beta-lactams. It was therefore hypothesized that peptidoglycan polymerization by these enzymes could underpin antibiotic resistance. Here, we investigated the catalytic activity of the three canonical Ldts encoded by *C. difficile* (Ldt_Cd1_, Ldt_Cd2_, and Ldt_Cd3_) *in vitro* and explored their contribution to growth and antibiotic resistance. We show that two of these enzymes catalyze the formation of novel types of peptidoglycan cross-links using *meso*-diaminopimelic acid both as a donor and an acceptor, also observed in peptidoglycan sacculi. We demonstrate that the simultaneous deletion of these three genes only has a minor impact on both peptidoglycan structure and resistance to beta-lactams. This unexpected result therefore implies that the formation of 3-3 peptidoglycan cross-links in *C. difficile* is catalyzed by as yet unidentified noncanonical Ldt enzymes.

*Clostridioides difficile* is a spore-forming Gram-positive obligate anaerobe that can cause hospital-associated diarrhea worldwide, representing increasing healthcare resource and economic burden (1). Although *C. difficile* has been recognized as a major cause of healthcare-associated infections since the 1970s, the more recent increase in morbidity and mortality is linked to the emergence of virulent epidemic strains including those belonging to ribotype 027 (2). *C. difficile* infections are underpinned by the natural resistance of this organism to several antibiotics including broad-spectrum beta-lactams such as cephalosporins. The dysbiosis caused by an antibiotic treatment creates an environment conducive to the germination of *C. difficile* spores and the production of virulence factors including toxins and several surface proteins (3).

The resistance of *C. difficile* to beta-lactams is poorly understood. These antibiotics covalently bind to D,D-transpeptidases (also known as penicillin-binding proteins) and irreversibly inhibit the enzymatic activity of these enzymes (4). In most bacteria, inhibition of D,D-transpeptidation disrupts the polymerization of peptidoglycan, the major and essential component of the bacterial cell wall and prevents bacterial growth (5). The peptidoglycan of *C. difficile* has an unusual composition. It is mostly polymerized by a class of enzymes called L,D transpeptidases (Ldts) (6). Unlike D,D-transpeptidases, which form bonds between the amino acids in positions 3 and 4 of peptidoglycan peptide stems (4-3 cross-links), Ldts form bonds between two amino acids in positions 3 (3-3 cross-links). The activity of Ldts involves a catalytic mechanism distinct from the mechanism of D,D-transpeptidases, and Ldts are not inhibited by beta-lactams, with the exception of penems and carbapenems (7). The *C. difficile* genome encodes three enzymes called Ldt_Cd1_, Ldt_Cd2_, and Ldt_Cd3_, which contain a canonical Ldt domain (YkuD). The contribution of these three enzymes to the peptidoglycan structure was investigated in strain 630 (6). Despite attempts to generate a triple knockout strain, only genes encoding Ldt_Cd1_ and Ldt_Cd2_ could be inactivated simultaneously (6) so it was suggested that 3-3 cross-links were required for viability. Analysis of the peptidoglycan structure in the double mutant strain revealed a limited decrease of 3-3 cross-links (6). The mutant remained able to perform 3-3 cross-links in the presence of ampicillin, suggesting that *C. difficile* Ldts were insensitive to this antibiotic (6). *In vitro* experiments revealed that these enzymes display distinct enzymatic activities and inhibition by beta-lactams (8). All enzymes were reported to have L,D carboxypeptidase activity, but L,D-transpeptidation and exchange of the amino acid in position 4 could only be detected for Ldt_Cd2_ and Ldt_Cd3_. Interestingly, Ldt_Cd3_ could not be acylated by any of the beta-lactams tested. The acylation efficacy of Ldt_Cd1_ and Ldt_Cd2_ by penicillin and cephalosporin antibiotics was much lower than the acylation by carbapenems, and the hydrolysis of these antibiotics was more efficient. It was therefore concluded that Ldt_Cd_ activity could only be inhibited by carbapenems (8).

Outstanding questions remain on the individual role of Ldt_Cd_ enzymes in peptidoglycan polymerization, the essentiality of the L,D-transpeptidation pathway in *C. difficile* and its contribution to antibiotic resistance. In this work, we further investigate the enzymatic activity of *C. difficile* Ldts, both *in vitro* and during vegetative growth. We show that Ldt_Cd2_ and Ldt_Cd3_ display novel enzymatic activities and that the genes encoding the three canonical Ldts can be deleted simultaneously. High-resolution structure of the wildtype and triple mutant peptidoglycan only revealed a minor impact on muropeptide composition, and no change in resistance to beta-lactams could be detected in the mutant strain. This work therefore provides new insights into the catalytic activities of Ldts and implies that the existence of another unidentified type of enzyme(s) that does not contain a canonical YkuD domain is able to catalyze the formation of 3-3 cross-links in *C. difficile*.

## Results

### *In vitro* assays with recombinant Ldt_Cd1_, Ldt_Cd2_, and Ldt_Cd3_ reveal distinct activities and a novel type of L,D-transpeptidation

**Figure 1:**
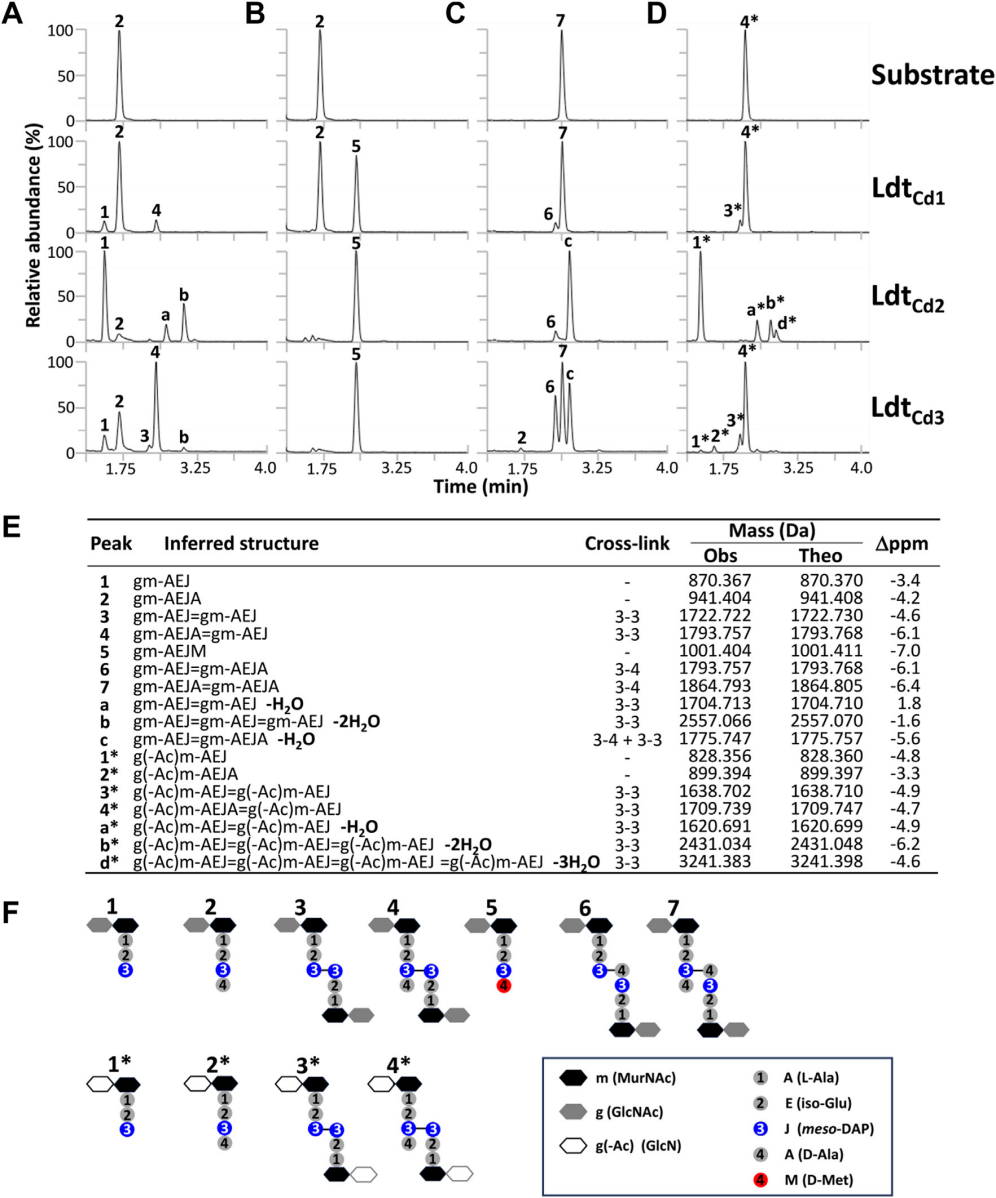
**HPLC-MS chromatograms of Ldt *in vitro* assays.** Recombinant enzymes were incubated in the presence of a disaccharide-tetrapeptide substrate to test carboxypeptidase and transpeptidase activity and exchange (*A* and *B*, respectively). Endopeptidase activity was tested using either a 4-3 cross-linked or a 3-3 cross-linked dimer (*C* and *D*, respectively). The inferred structures from LC-MS analysis (*E*) as well as expected structures (*F*) are described. All traces correspond to Total Ion Chromatograms (LC-MS data) corresponding to average intensity values from three independent experiments. The difference between observed and theoretical masses (Δppm) was calculated ((Theoretical mass – Observed mass)/Theoretical mass∗10E6).

We sought to investigate the activities of the three Ldt_Cd_ enzymes to identify their specific roles in peptidoglycan remodeling. The recombinant enzymes were purified (Fig. S1) to test their enzymatic activities using four types of purified substrates: (i) a disaccharide-tetrapeptide alone (GlcNAc-MurNAc-L-Ala-D-isoGlu-*meso*-DAP-D-Ala; gm-AEJA, where “J” represents *meso*-diaminopimelic acid [DAP]) to test L,D-carboxypeptidase and L,D-transpeptidase activities (Fig. 1*A*); (ii) the same disaccharide-tetrapeptide (gm-AEJA) in the presence of D-methionine to test fourth amino acid exchange (Fig. 1*B*); (iii) a 4-3 cross-linked dimer ((GlcNAc-MurNAc-L-Ala-D-isoGlu-*meso*-DAP-D-Ala)_2_; gm-AEJA=gm-AEJA, where “=” represents a peptidoglycan cross-link) (Fig. 1*C*); and (iv) a 3-3 cross-linked dimer (GlcN-MurNAc-L-Ala-D-isoGlu-*meso*-DAP-D-Ala-GlcN-MurNAc-L-Ala-D-isoGlu-*meso*-DAP (g(-Ac)m-AEJA=g(-Ac)m-AEJA) to test endopeptidase activities ((Fig. 1*D*). The monomer and 4-3 cross-linked dimer were purified from the *Escherichia coli* Δ6*ldt* strain and therefore contain fully acetylated sugars (9). The 3-3 cross-linked dimer was purified from *C. difficile* and therefore contained deacetylated GlcNAc (GlcN).

Ldt_Cd_ recombinant enzymes were active against all substrates tested and revealed distinct preferential activities. Ldt_Cd1_ displayed a low carboxypeptidase and transpeptidase activity and only converted half of the substrate during the exchange reaction. No endopeptidase activity was detected with any of the dimers.

Ldt_Cd2_ had a preferential carboxypeptidase activity on the gm-AEJA substrate that was mostly converted into a disaccharide-tripeptide (gm-AEJ) and transformed all the monomer into gm-AEJM. A very weak carboxypeptidase activity was detected with the 4-3 dimer while all the 3-3 dimers were completely cleaved, releasing disaccharide-tripeptides (g(-Ac)m-AEJ) as the most abundant products. Ldt_Cd2_ was only active on 3-3 cross-linked dimers. Interestingly, several multimers matching the expected mass for dimers, trimers, and tetramers lacking a molecule of water were detected (labeled as a, b, c, a∗, b∗ and d∗; Fig. 1*E*). These were further analyzed by NMR.

Ldt_Cd3_ had the highest L,D-transpeptidase activity of all enzymes, and the 3-3 cross-linked dimer was the most abundant product generated from the gm-AEJA substrate. This enzyme also displayed some carboxypeptidase activity, using the monomer or both dimers and converted all the monomer into gm-AEJM. Surprisingly, the carboxypeptidase activity of Ldt_Cd3_ was higher on the 4-3 dimer than on the 3-3 dimer. Ldt_Cd3_ also produced two transpeptidation products matching the mass of a 3-3 dimer lacking a molecule of water (peak b, also detected with Ldt_Cd2_) and the mass of a 4-3 dimer lacking a molecule of water (peak c). The structures of all expected and previously described muropeptides produced by Ldt_Cd1_, Ldt_Cd2_, and Ldt_Cd3_ are described in Figure 1*E*.

### Tandem mass spectrometry and NMR analyses of Ldt_Cd2_ and Ldt_Cd3_ transpeptidation products reveal a novel type of peptidoglycan cross-links

**Figure 2:**
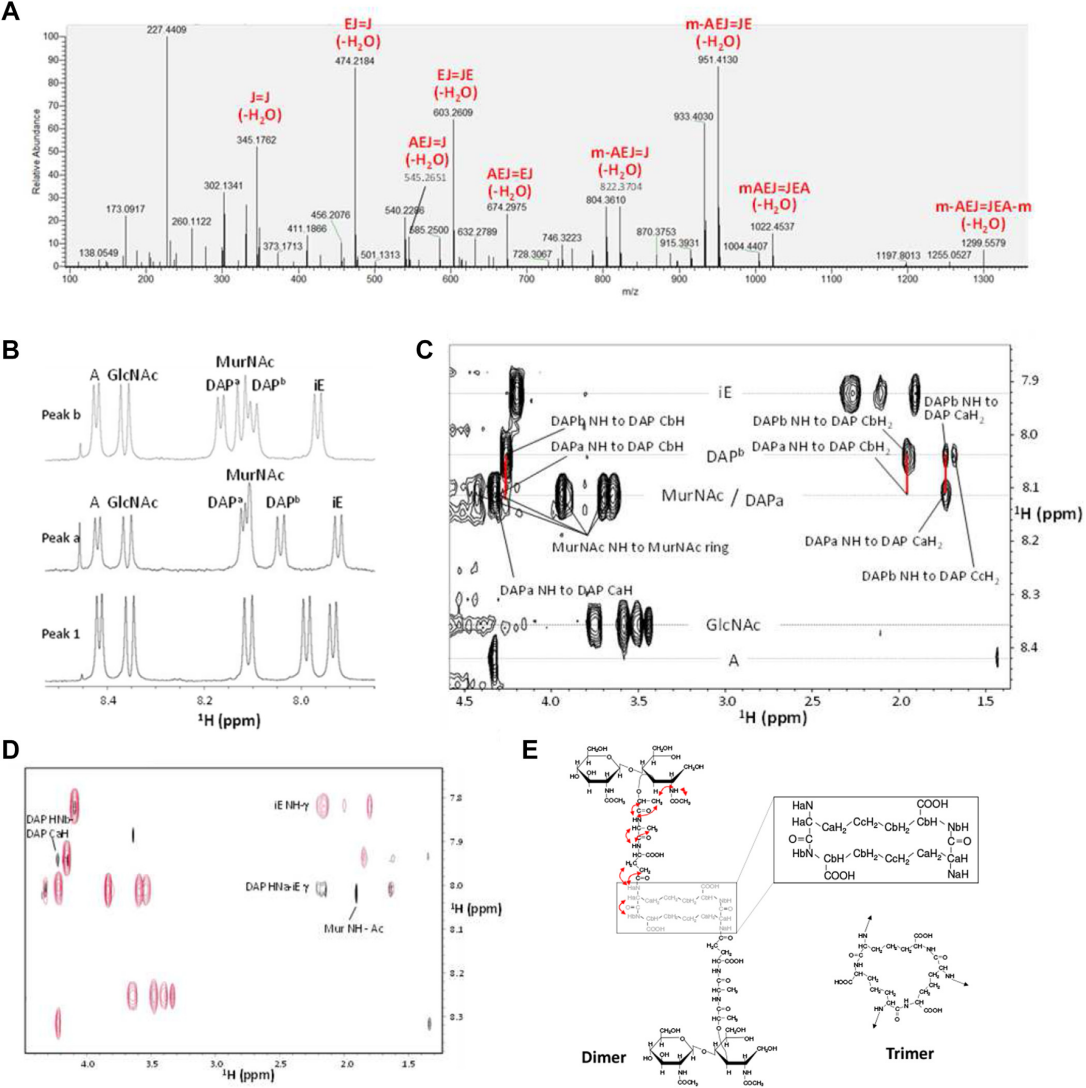
**MS/MS and NMR analysis of unusual muropeptides a and b.***A*, MS/MS analysis of the ion corresponding to peak a (*m/z* = 1705.7211). Nine fragment ions corresponding to peptides with doubly bonded m-DAP residues are indicated. *B*, 1D NMR spectra of peptidoglycan fragments identified during *in vitro* assays (see Fig. 1): peak 1 (gm-AEJ) was used as a control; peak a corresponds to a dimer (gm-AEJ=gm-AEJ) with an unusual cross-link; peak b corresponds to a trimer (gm-AEJ=gm-AEJ=gm-AEJ) with an unusual cross-link The identity of each amide proton is indicated on the spectra. *C*, part of the TOCSY spectrum of the muropeptide in peak a, showing connectivities between amide protons and side chains. The signals linked by *red lines* are the connectivities for the DAP^a^ and DAP^b^ amides, showing that they connect to identical side-chain frequencies and are therefore part of the same spin system. *D*, TOCSY (*red*) and NOESY (*black*) spectra of the dimer. Significant peaks are marked. *E*, structures of (*left*) dimer (with NOEs indicated) and (*right*) trimer. Only the central part of the trimer is shown, with arrows indicating where the AEJ chains are attached. A, alanine; DAP^a^, *meso*-diaminopimelic acid backbone (directly bonded to the isoglutamate); DAP^b^, *meso*-diaminopimelic acid side chain; GlcNAc, *N*-acetylglucosamine; iE, isoglutamate; MurNAc, *N*-acetylmuramic acid.

The muropeptide contained in peak a (Fig. 1) was analyzed by tandem mass spectrometry (MS/MS). The Fragmentation spectrum confirmed the inferred structure for a dimer with doubly cross-linked *meso-*DAP residues used both as an acceptor and as a donor group (Fig. 2*A*; see ions labeled). Several signature ions were found, including a doubly cross-linked DAP–DAP fragment.

Peptidoglycan fragments in peaks 1, a, and b (Fig. 1*E*) were purified and further analyzed by NMR. One-dimensional (1D) NMR spectra of the peptidoglycan fragments demonstrated a high purity for each. The monomer gm-AEJ (peak 1) used as a control (Fig. 2*B*, bottom panel) had all the amide signals expected, namely, two sugar *N*-acetyl signals, and one signal each for the Ala, DAP, and isoGlu (iE) residues. There is only one signal for DAP because one amine forms an amide with iE, while the other is a free amino group and therefore exchanges too fast with water to be visible. All the other signals are as expected, including the presence of two *N*-acetyl methyl singlets from the two sugars and two methyl doublets from Ala and the lactyl group on MurNAc.

The dimer corresponding to peak a has a remarkably simple NMR spectrum (Fig. 2*B*, middle panel). The amide region contains only six amide doublets, two *N*-acetyl methyl singlets, and two methyl doublets, as seen in the monomer (Fig. S2). This simplicity very strongly suggests a symmetrical dimer, and the similarity of the amide chemical shifts between the monomer and dimer implies a similar covalent structure.

**Figure 3:**
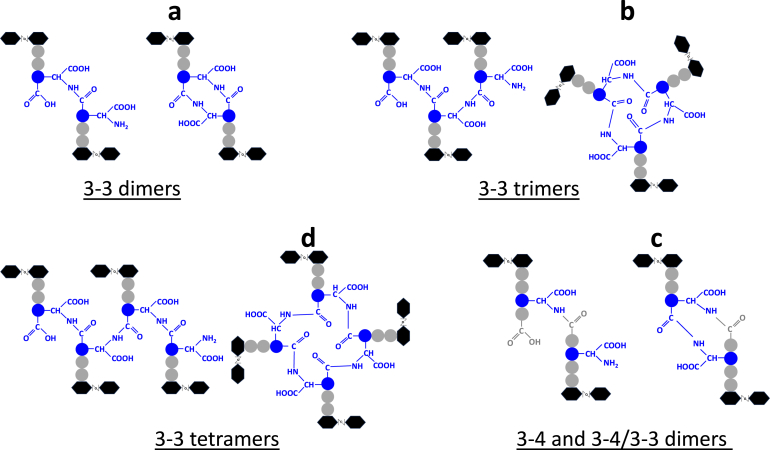
**Unusual peptidoglycan cross-links catalyzed by Ldt**_**Cd2**_**and Ldt**_**Cd3**_**.** Based on NMR data, the structure of muropeptides corresponding to peaks a, b, c, and d are shown next to the structure of the canonical 3-3 or 4-3 dimers, 3-3 trimer, and 3-3 tetramer. Dimer c contains both types of cross-links (3-3 and 4-3), resulting from D,D- and L,D-transpeptidation. Muropeptides a∗, b∗, d∗ display the same cross-links as a, b, d but contain GlcN instead of GlcNAc.

There are two amide signals from the diaminopimelate, visible in the total correlation spectroscopy (TOCSY) spectrum because they belong to the same spin system (Fig. 2*C*), indicating that both amines in the diaminopimelate take part in amide bonds. Chemical shift assignments for the dimer are listed in Table S1. In the nuclear Overhauser enhancement spectroscopy (NOESY) spectrum, there are the expected sequential NOEs present between NH_*i*_ and protons in residue (*i*-1), as indicated in Figure 2*D*. Crucially, these include NOEs between DAP NH^a^ and iE CγH_2_, and the other “sequential” NOE of DAP NH^b^ to DAP CaH (Fig. 2, *D* and *E*). Similarly, the spectrum of trimer b (Fig. 2*B*, top panel) is also very similar. The chemical shifts remain very similar to the monomer and dimer, and again there is only one set of signals, indicating a symmetrical trimer. The NMR spectra are thus fully consistent with the structures described in Figure 3, and the simplicity of the spectrum means that no unsymmetrical structure is possible. Based on our NMR data, we conclude that the muropeptides in peaks a, b, c, and d all correspond to multiply cross-linked structures (Figs. 2 and 3).

### TraDIS data mining and gene deletion reveal that the three canonical *C. difficile* Ldts are nonessential

**Figure 4:**
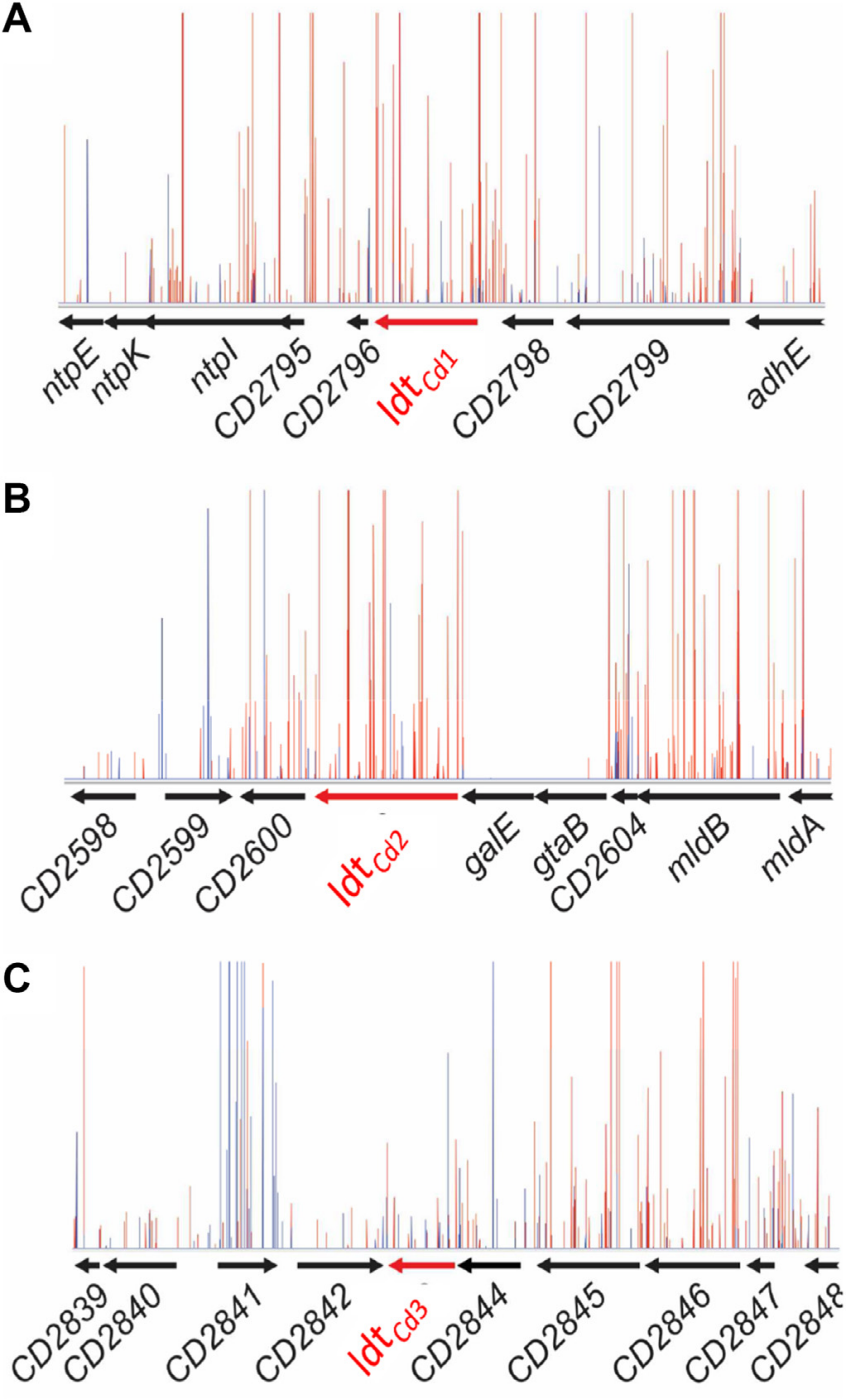
**TraDIS analysis of the ldtCd loci.** The number of transposition events in ldtCd1 (*A*), ldtCd2 (*B*), and ldtCd3 (*C*) are shown as histograms depicting the localization (*x*-axis) and the frequency (*y*-axis) of transposon insertion sites (in *red* are antisense insertions; in *blue* are sense insertions).

Previous attempts to build a mutant with deletions in all three genes encoding the canonical Ldts were unsuccessful (6), suggesting either that one of them is essential or that 3-3 cross-linking is essential. We took advantage of transposon-directed insertion site sequencing (TraDIS) data previously published in a study that identified essential genes in *C. difficile* R20291 (10). Using the number of transposon insertions in each *ldt*_*Cd*_ gene as a proxy to determine essentiality, we concluded that none of these genes was essential (Fig. 4), leaving the possibility that the combined deletion of *ldt*_*Cd1*_, *ldt*_*Cd2*_, and *ldt*_*Cd3*_ could be non viable. To test this hypothesis, we sought to generate a series of in-frame deletions in *ldt*_*Cd1*_*, ldt*_*Cd2*_, and *ldt*_*Cd3*_. All genes were deleted individually or simultaneously. All the combinations of deletions, including the triple deletion mutant, could be obtained, showing that these genes are not required for viability. Since this result was unexpected, we performed whole genome sequencing on the mutants and confirmed the deletion of the *ldt* genes in the strains sequenced. Single nucleotide polymorphism analysis identified a unique single mutation in the triple deletion mutant (File S1 and Fig. S3). The mutation (T>TA) occurred at position 581480 in the intergenic mutation between *CD0482* and *glsA*, two genes with no known link to peptidoglycan polymerization, encoding a putative phosphoribulokinase/uridine kinase and a putative glutaminase, respectively. This result therefore suggests that the deletion of the *ldt*_*Cd*_ genes does not lead to genetic mutations likely to compensate for the lack of L,D-transpeptidase activity.

### High-resolution structure of the wildtype and triple mutant peptidoglycans

**Figure 5:**
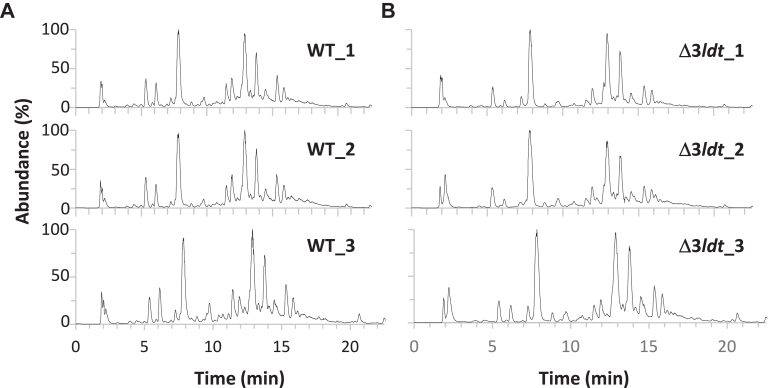
**HPLC-MS chromatogram of *C. difficile* reduced disaccharide-peptides.** Each total ion chromatogram corresponds to a biological replicate of strain R20291 (WT) (*A*) and its isogenic derivative with in-frame deletions in genes *ldt*_*Cd1*_, *ldt*_*Cd2*_, and *ldt*_*Cd3*_ (Δ3*ldt*) (*B*).

Peptidoglycan was extracted from vegetative cells in stationary phase, and soluble fragments released after mutanolysin digestion were analyzed by liquid chromatography coupled to tandem mass spectrometry (LC-MS/MS). Surprisingly, the chromatograms of the WT and triple mutant were virtually identical, indicating a very minor contribution of the three Ldts to peptidoglycan structure (Fig. 5).

To investigate subtle differences associated with the simultaneous deletion of the three *ldt*_*Cd*_ genes, we performed a high-resolution analysis of the LC-MS/MS datasets using the Byos (Protein Metrics by Dotmatics) and PGFinder software (11). A bespoke search strategy was designed (Fig. S4). A first search was performed to identify the monomer search space using the Byonic module from the proprietary software Byos. Thirty-four disaccharide-peptides with a fragmentation showing more than half of the expected *b* and *y* ions were identified (Fig. S5). These included 24 deacetylated monomers containing di-, tri-, tetra-, and pentapeptide stems (g(-Ac)m-AE, g(-Ac)m-AEJ, g(-Ac)m-AEJX, and g(-Ac)m-AEJAX) and 10 fully acetylated monomers (gm-AE, gm-AEJ, gm-AEJX, and gm-AEJAX), where X can be any amino acid. A database called DB_0 made of these 34 monomers was used to perform a PGFinder search (step 2 in Fig. S4) to identify the most abundant monomers. Thirteen disaccharide-peptides accounting for more than 98% of the monomers identified were selected to create a second database (DB_1) containing dimers resulting from 3-3 and 4-3 cross-linking. A third PGFinder search (step 3 in Fig. S4) was performed to identify the most abundant dimers and generate the next database containing monomers, dimers, and trimers (DB_2). The next search with PGFinder and DB_2 (step 4 in Fig. S4) identified the most abundant trimers. A final database called DB_3 was created using all the information from sequential searches; it contained all MS/MS-checked monomers, 26 dimers, 16 trimers, and all AnhydroMurNAc derivatives of the 10 most abundant mono-, di-, and trimers, as well as 4 unusually cross-linked structures identified during *in vitro* assays.

The result of the PGFinder search using DB_3 and biological replicates from the WT and triple mutant is described in Table 1. The search strategy described here combining both LC-MS and LC-MS/MS analysis allowed us to identify 97 muropeptides, which is an unprecedentedly detailed analysis. The comparison between the two strains revealed a remarkable similarity between the two peptidoglycan compositions (Table 2). No significant difference was found when comparing the proportion of monomers, dimers, trimers, or glycan chain length. Cross-linking index as well as the proportion of 3-3 cross-links was also similar. The only difference found was a significant decrease in the exchange reaction (23.4 ± 0.7% in the WT and 16.9 ± 1.4% in the triple mutant). Overall, our analysis therefore demonstrated that the three canonical L,D-transpeptidases Ldt_Cd1_, Ldt_Cd2_, and Ldt_Cd3_ only contribute marginally to remodel the peptidoglycan of *C. difficile* vegetative cells.

**Table 1:** Muropeptide analysis of *C. difficile* WT and triple *ldt*_*Cd1*_, *ldt*_*Cd2*_, *ldt*_*Cd3*_ mutant (Δ3*ldt*)

Muropeptide^a^	Ldt activity/crosslink^b^	Abundance (%)^c^		RT (min)	Average^d^ Δppm
		WT	Δ*3ldt*		
gm(-Ac)-AEJA|1	N/A	21.594% ± 1.050%	28.192% ± 4.671%	7.90 ± 0.02	2.71
gm(-Ac)-AEJ|1	Carboxypeptidase	9.002% ± 1.348%	8.235% ± 1.026%	5.39 ± 0.03	2.85
gm(-Ac)-AEJG|1	Exchange	9.814% ± 0.907%	4.696% ± 1.177%	6.16 ± 0.02	2.55
gm(-Ac)-AEJF|1	Exchange	1.820% ± 0.806%	2.083% ± 0.886%	20.72 ± 0.03	2.14
gm(-Ac)-AE|1	N/A	3.174% ± 0.185%	2.297% ± 0.520%	7.74 ± 0.05	2.31
gm-AEJA|1	N/A	0.512% ± 0.061%	0.716% ± 0.211%	8.84 ± 0.02	1.50
gm(-Ac)-AEJV|1	Exchange	0.944% ± 0.111%	0.570% ± 0.113%	13.83 ± 0.02	1.71
gm(-Ac)-AEJI|1	Exchange	0.294% ± 0.076%	0.389% ± 0.131%	18.20 ± 0.04	0.66
gm(-Ac)-AEJY|1	Exchange	0.172%	0.328% 0.072%	15.82 ± 0.04	0.99
gm(-Ac)-AEJAA|1	N/A	0.244% ± 0.007%	0.311% ± 0.053%	9.63 ± 0.08	1.68
gm-AEJA (Anh)|1	N/A	0.168% ± 0.010%	0.270% ± 0.085%	7.97 ± 0.11	2.24
gm(-Ac)-AEJS|1	Exchange	0.302% ± 0.036%	0.234% ± 0.039%	5.52 ± 0.02	2.04
gm-AEJ|1	N/A	0.197% ± 0.025%	0.207% ± 0.130%	9.41 ± 4.13	1.89
gm(-Ac)-AEJK|1	Exchange	0.324% ± 0.086%	0.184% ± 0.053%	4.55 ± 0.06	2.08
gm(-Ac)-AEJQ|1, gm(-Ac)-AEJAG|1	Exchange	0.107% ± 0.011%	0.095% ± 0.022%	8.41 ± 1.39	0.86
gm(-Ac)-AEJA (Anh)|1	N/A	0.033% ± 0.002%	0.084% ± 0.010%	11.35 ± 0.02	0.31
gm-AEJG|1	Exchange	0.115% ± 0.026%	0.071% ± 0.010%	7.74 ± 0.75	1.08
gm-AE|1	N/A	0.081% ± 0.016%	0.062% ± 0.019%	8.65 ± 0.03	0.41
gm(-Ac)-AEJAT|1	N/A	0.177% ± 0.021%	0.096% ± 0.022%	6.63 ± 0.04	0.61
gm(-Ac)-AEJAF|1	N/A	0.042% ± 0.015%	0.055% ± 0.018%	24.36 ± 0.02	1.30
gm-AEJI|1	Exchange	0.015% ± 0.009%	0.042% ± 0.028%	16.08 ± 4.43	0.88
gm(-Ac)-AEJN|1	Exchange	0.235% ± 0.065%	0.062% ± 0.004%	5.05 ± 0.03	1.49
gm(-Ac)-AEJH|1	Exchange	0.104% ± 0.080%	ND	5.19 ± 0.20	2.01
gm(-Ac)-AEJE|1	Exchange	0.062% ± 0.010%	0.041% ± 0.003%	7.51 ± 0.03	1.37
gm-AEJF|1	Exchange	0.019% ± 0.013%	0.020% ± 0.014%	21.95 ± 0.01	1.35
gm-AEJK|1, gm(-Ac)-AEJAV|1 (mixture)	Exchange	0.000% ± 0.000%	0.000% ± 0.000%	6.69 ± 3.18	1.88
gm(-Ac)-AEJAI|1	N/A	0.021% ± 0.003%	0.023% ± 0.003%	19.36 ± 0.02	0.76
gm(-Ac)-AEJAK|1	N/A	0.019% ± 0.001%	0.024% ± 0.006%	7.22 ± 0.12	1.79
gm(-Ac)-AEJD|1	N/A	0.125% ± 0.027%	0.024% ± 0.004%	6.27 ± 0.04	2.03
gm-AEJH|1	Exchange	0.014% ± 0.007%	ND	6.40 ± 0.02	0.74
gm(-Ac)-AEJT|1	Exchange	0.025% ± 0.002%	0.020% ± 0.002%	6.49 ± 0.14	1.08
gm(-Ac)-AE (Anh)|1	N/A	0.029% ± 0.004%	0.015% ± 0.004%	12.00 ± 0.01	1.58
gm-AEJAA|1	N/A	0.010% ± 0.001%	0.010% ± 0.000%	10.79 ± 1.54	1.10
gm(-Ac)-AEJ (Anh)|1	N/A	0.025% ± 0.005%	0.013% ± 0.003%	8.56 ± 0.01	0.58
gm(-Ac)-AEJAY|1	N/A	0.005%	0.005% ± 0.004%	17.18 ± 0.03	0.22
gm(-Ac)-AEJG (Anh)|1	Exchange	0.015% ± 0.004%	0.006% ± 0.003%	9.31 ± 0.10	0.46
gm(-Ac)-AEJAA (Anh)|1	Exchange	ND	0.001% ± 0.000%	9.49 ± 0.04	1.74
gm(-Ac)-AEJY (Anh)|1	Exchange	ND	0.000%	24.42 ± 0.03	1.39
gm-AEJAF|1	N/A	0.001% ± 0.001%	0.001% ± 0.001%	24.07 ± 0.01	1.04
gm(-Ac)-AEJA=gm(-Ac)-AEJ|2	3-3	16.323% ± 0.074%	19.316% ± 1.031%	13.09 ± 0.08	1.31
gm(-Ac)-AEJA=gm(-Ac)-AEJA|2	3-4	6.806% ± 1.727%	8.963% ± 1.708%	13.93 ± 0.11	1.20
gm(-Ac)-AEJ=gm(-Ac)-AEJ|2	3-3	5.903% ± 1.156%	5.446% ± 0.636%	11.99 ± 0.03	1.57
gm(-Ac)-AEJG=gm(-Ac)-AEJ|2	3-3	5.741% ± 0.730%	2.688% ± 0.881%	11.54 ± 0.04	1.39
gm-AEJA=gm(-Ac)-AEJ|2	3-3	1.238% ± 0.141%	1.677% ± 0.398%	13.80 ± 0.03	1.77
gm(-Ac)-AEJF=gm(-Ac)-AEJ|2	3-3	0.682% ± 0.213%	0.719% ± 0.283%	22.56 ± 0.01	1.85
gm-AEJA=gm(-Ac)-AEJA|2	3-4	0.560% ± 0.052%	0.713% ± 0.151%	14.78 ± 0.08	0.91
gm(-Ac)-AEJG=gm(-Ac)-AEJA|2	3-4	1.112% ± 0.202%	0.467% ± 0.114%	12.40 ± 0.02	0.68
gm-AEJA=gm(-Ac)-AEJ (Anh)|2	3-3	0.244% ± 0.036%	0.374% ± 0.131%	19.22 ± 0.10	0.83
gm(-Ac)-AEJV=gm(-Ac)-AEJ|2	3-3	0.308% ± 0.047%	0.205% ± 0.057%	17.77 ± 0.02	0.57
gm(-Ac)-AEJA=gm(-Ac)-AEJ (Anh)|2	3-3	0.337% ± 0.040%	0.244% ± 0.023%	15.68 ± 0.32	1.47
gm(-Ac)-AEJI=gm(-Ac)-AEJ|2	3-3	0.138% ± 0.055%	0.176% ± 0.073%	20.98 ± 0.02	0.88
gm-AEJ=gm(-Ac)-AEJ|2	3-3	0.250% ± 0.011%	0.250% ± 0.092%	12.69 ± 0.03	1.26
gm(-Ac)-AEJA=gm(-Ac)-AEJA (Anh)|2	3-4	0.113% ± 0.007%	0.176% ± 0.011%	16.99 ± 0.02	0.34
gm(-Ac)-AEJF=gm(-Ac)-AEJA|2	3-4	0.161% ± 0.061%	0.130% ± 0.043%	23.14 ± 0.02	1.43
gm(-Ac)-AEJY=gm(-Ac)-AEJ|2	3-3	0.063% ± 0.084%	0.107% ± 0.034%	18.74 ± 1.18	0.37
gm(-Ac)-AEJS=gm(-Ac)-AEJ|2	3-3	0.207% ± 0.005%	0.133% ± 0.014%	11.41 ± 0.14	0.63
gm-AEJA=gm(-Ac)-AEJA (Anh)|2	3-4	0.072% ± 0.019%	0.125% ± 0.012%	14.02 ± 0.30	9.40
gm(-Ac)-AEJK=gm(-Ac)-AEJ|2	3-3	0.154% ± 0.033%	0.080% ± 0.022%	10.20 ± 0.02	0.27
gm(-Ac)-AEJI=gm(-Ac)-AEJA|2	3-4	0.037% ± 0.013%	0.042% ± 0.016%	21.78 ± 0.02	0.42
gm(-Ac)-AEJV=gm(-Ac)-AEJA|2	3-4	0.067% ± 0.006%	0.038% ± 0.011%	18.60 ± 0.02	0.35
gm(-Ac)-AEJS=gm(-Ac)-AEJA|2	3-4	0.053% ± 0.006%	0.046% ± 0.008%	12.09 ± 0.02	1.10
gm(-Ac)-AEJAA=gm(-Ac)-AEJA|2	3-4	0.013% ± 0.006%	0.027% ± 0.002%	13.96 ± 0.51	0.48
gm(-Ac)-AEJ=gm(-Ac)-AEJ (Anh)|2	3-3	0.110% ± 0.021%	0.011%	14.84 ± 0.22	0.30
gm(-Ac)-AEJN=gm(-Ac)-AEJ|2	3-3	0.094% ± 0.025%	0.028% ± 0.005%	10.88 ± 0.02	0.64
gm(-Ac)-AEJK=gm(-Ac)-AEJA|2	3-4	0.044% ± 0.008%	0.027% ± 0.002%	10.97 ± 0.02	0.56
gm(-Ac)-AEJY=gm(-Ac)-AEJA|2	3-4	0.013% ± 0.000%	0.021% ± 0.005%	18.92 ± 0.02	0.31
gm(-Ac)-AEJA=gm(-Ac)-AEJ (-H2O)|2	3-3∗	0.138% ± 0.000%	0.016% ± 0.005%	14.67 ± 0.54	0.55
gm(-Ac)-AEJN=gm(-Ac)-AEJA|2	3-4	0.021% ± 0.006%	0.009% ± 0.001%	11.62 ± 0.02	1.08
gm(-Ac)-AEJG=gm(-Ac)-AEJA (Anh)|2	3-4	0.004%	0.000%	15.38 ± 0.00	2.26
gm(-Ac)-AEJF=gm(-Ac)-AEJ (Anh)|2	3-3	0.010% ± 0.002%	0.014% ± 0.014%	20.96 ± 4.59	5.28
gm(-Ac)-AEJG=gm(-Ac)-AEJ (Anh)|2	3-3	0.126% ± 0.017%	0.001%	15.56 ± 2.77	0.79
gm(-Ac)-AEJI=gm(-Ac)-AEJ (Anh)|2	3-3	0.002% ± 0.001%	0.001% ± 0.000%	23.32 ± 0.03	1.04
gm(-Ac)-AEJV=gm(-Ac)-AEJ (Anh)|2	3-3	0.015% ± 0.011%	0.001%	19.18 ± 3.13	2.32
gm(-Ac)-AEJA=gm(-Ac)-AEJ=gm(-Ac)-AEJ|3	3-3	3.428% ± 0.114%	3.041% ± 0.233%	15.39 ± 0.04	1.42
gm(-Ac)-AEJA=gm(-Ac)-AEJA=gm(-Ac)-AEJ|3	3-3, 3-4	1.823% ± 0.087%	2.273% ± 0.327%	15.98 ± 0.02	1.09
gm-AEJA=gm(-Ac)-AEJA=gm(-Ac)-AEJ|3	3-3, 3-4	0.344% ± 0.047%	0.446% ± 0.133%	16.76 ± 0.02	0.27
gm-AEJA=gm(-Ac)-AEJ=gm(-Ac)-AEJ|3	3-3	0.505% ± 0.052%	0.449% ± 0.135%	16.19 ± 0.02	0.48
gm(-Ac)-AEJA=gm(-Ac)-AEJA=gm(-Ac)-AEJA|3	3-4	0.348% ± 0.040%	0.468% ± 0.060%	16.55 ± 0.03	0.30
gm-AEJA=gm(-Ac)-AEJ=gm(-Ac)-AEJ (Anh)|3	3-3	0.259% ± 0.026%	0.342% ± 0.073%	20.34 ± 0.03	1.20
gm(-Ac)-AEJ=gm(-Ac)-AEJ=gm(-Ac)-AEJ|3	3-3	0.638% ± 0.165%	0.395% ± 0.046%	14.46 ± 0.02	0.48
gm(-Ac)-AEJG=gm(-Ac)-AEJ=gm(-Ac)-AEJ|3	3-3	0.565% ± 0.090%	0.194% ± 0.068%	14.13 ± 0.02	0.23
gm-AEJA=gm(-Ac)-AEJA=gm(-Ac)-AEJA|3	3-4	0.095% ± 0.015%	0.142% ± 0.032%	17.30 ± 0.02	0.31
gm(-Ac)-AEJG=gm(-Ac)-AEJA=gm(-Ac)-AEJ|3	3-3, 3-4	0.253% ± 0.052%	0.115% ± 0.038%	14.69 ± 0.01	0.31
gm-AEJA=gm(-Ac)-AEJA=gm(-Ac)-AEJ (Anh)|3	3-3, 3-4	0.097% ± 0.008%	0.136% ± 0.017%	20.18 ± 1.58	2.32
gm(-Ac)-AEJF=gm(-Ac)-AEJ=gm(-Ac)-AEJ|3	3-3	0.061% ± 0.018%	0.041% ± 0.019%	23.03 ± 0.01	1.05
gm(-Ac)-AEJA=gm(-Ac)-AEJ=gm(-Ac)-AEJ (Anh)|3	3-3	0.174% ± 0.019%	0.049% ± 0.008%	17.55 ± 0.16	0.30
gm(-Ac)-AEJA=gm(-Ac)-AEJA=gm(-Ac)-AEJ (Anh)|3	3-3, 3-4	0.062% ± 0.007%	0.051% ± 0.005%	18.40 ± 0.09	0.40
gm(-Ac)-AEJF=gm(-Ac)-AEJ=gm(-Ac)-AEJA|3	3-3, 3-4	0.036% ± 0.013%	0.026% ± 0.010%	23.39 ± 0.02	0.40
gm(-Ac)-AEJG=gm(-Ac)-AEJA=gm(-Ac)-AEJA|3	3-4	0.047% ± 0.012%	0.021% ± 0.008%	15.23 ± 0.01	0.59
gm(-Ac)-AEJG=gm(-Ac)-AEJ=gm(-Ac)-AEJ (Anh)|3	3-3	0.047% ± 0.007%	ND	16.24 ± 0.02	0.22
gm(-Ac)-AEJ=gm(-Ac)-AEJ=gm(-Ac)-AEJ (-H2O)|3	3-3∗	0.056% ± 0.015%	ND	16.98 ± 0.03	1.18
gm(-Ac)-AEJA=gm(-Ac)-AEJA=gm(-Ac)-AEJA (Anh)|3	3-4	0.013% ± 0.001%	0.017% ± 0.002%	19.02 ± 0.04	0.40
gm(-Ac)-AEJG=gm(-Ac)-AEJ=gm(-Ac)-AEJA (Anh)|3	3-3, 3-4	0.011% ± 0.001%	ND	16.82 ± 0.12	0.25
gm(-Ac)-AEJA=gm(-Ac)-AEJ=gm(-Ac)-AEJ (-H2O)|3	3-3	0.002%	ND	16.83 ± 0.00	2.64
gm-AEJA=gm(-Ac)-AEJA=gm(-Ac)-AEJA (Anh)|3	3-4	0.004% ± 0.006%	0.016% ± 0.007%	17.35 ± 1.74	4.89
gm(-Ac)-AEJ=gm(-Ac)-AEJ=gm(-Ac)-AEJ (Anh)|3	3-3	0.083% ± 0.006%	0.005% ± 0.003%	16.68 ± 0.28	1.56
gm(-Ac)-AEJF=gm(-Ac)-AEJ=gm(-Ac)-AEJ (Anh)|3	3-3	0.002% ± 0.000%	0.005% ± 0.005%	23.51 ± 3.39	3.09
gm(-Ac)-AEJ=gm(-Ac)-AEJ=gm(-Ac)-AEJ (Anh)|3	3-3	0.083% ± 0.006%	0.005% ± 0.003%	16.68 ± 0.28	1.56
gm(-Ac)-AEJF=gm(-Ac)-AEJ=gm(-Ac)-AEJ (Anh)|3	3-3	0.002% ± 0.000%	0.005% ± 0.005%	23.51 ± 3.39	3.09

Abbreviation: RT, retention time.

a When multiple dimer structures are possible, the most likely structure is proposed based on the abundance of acceptor stems.

b N/A, not applicable; 3-3∗ crosslinks correspond to L,D-transpeptidation products doubly crosslinked.

c ND, not detected; no standard deviation is provided when muropeptides were only identified in a single replicate.

d Absolute mass difference between observed and theoretical mass in parts per million (ppm).

**Table 2:** Summary of WT and 3Δ*ldt* PG properties

**Muropeptides/properties**	WT	Δ3*ldt*
Monomers	49.93% ± 2.82%	49.96% ± 3.13%
Dimers	41.12% ± 2.15%	41.94% ± 2.19%
Trimers	8.95% ± 1.00%	8.09% ± 1.04%
Cross-linking index	23.51% ± 1.40%	23.64% ± 1.44%
Glycan chain length	96.6 ± 1.8	87.3 ± 18.7
gm-AEJX	23.39% ± 0.70%	16.86% ± 1.41%
3-3 cross-links	81.33% ± 2.54%	78.35% ± 2.68%

### Comparative phenomics of the parental R20291 strain and its isogenic Δ3*ldt* derivative

A comprehensive set of experiments were carried out to compare the phenotype of the R20291 strain and the triple *ldt*_*Cd*_ mutant. As expected, based on the results from peptidoglycan analysis, no significant differences were observed between the two strains in cell size (Fig. S6), sporulation (Fig. S7), or toxin release (Fig. S8). Minimum inhibitory concentrations (MICs) for several beta-lactams were also tested for all the mutants generated in this study and did not reveal any difference in the resistance against any of these antibiotics (Table S2).

## Discussion

Recombinant L,D-transpeptidases represent a class of enzymes that are amenable to study *in vitro* since they can use soluble peptidoglycan fragments as a substrate. *In vitro* assays with distinct peptidoglycan fragments purified from intact sacculi were used to explore the catalytic activities of the three *C. difficile* Ldts. Our comparative analysis based on an LC-MS/MS assay with several substrates provided information about the preferential activity of each Ldt. Previous studies reported that Ldt_Cd1_ only displayed carboxypeptidase activity (8). Our data confirmed this result and also revealed that it can also perform transpeptidation and exchange reactions, even though this enzyme was poorly active on all substrates tested. Ldt_Cd2_ was able to perform all reactions but preferentially acted as a carboxypeptidase. Remarkably, Ldt_Cd2_ was the only enzyme with endopeptidase activity, exclusively using 3-3 dimers as a substrate. Unlike other endopeptidases that cleave 3-3 cross-links (MepA, MepM, and MepK), Ldt_Cd2_ has a strict substrate specificity for 3-3 cross-links since no activity against 4-3 cross-links could be detected (12, 13) (Table S3). Ldt_Cd2_ activity therefore appears to be unique since it is the first enzyme described that is only active on 3-3 cross-links.

Ldt_Cd3_ displayed the highest transpeptidase/exchange activity and a relatively weak carboxypeptidase and endopeptidase activity, preferentially against 3-3 cross-linked dimers. Unlike Ldt_Cd2_, Ldt_Cd3_ could cleave 4-3 dimers with low efficiency.

Besides the exhaustive description of expected Ldt_Cd_ activities, our *in vitro* assays also revealed that Ldt_Cd2_ and Ldt_Cd3_ can generate a novel type of peptidoglycan cross-links. These result from double transpeptidation reactions that use meso-DAP both as a donor and an acceptor group. Interestingly, we identified double cross-linked dimers containing either two 3-3 cross-links or a mixture of 4-3 and 3-3 cross-links. In hindsight, this result is not entirely surprising since the catalytic reaction leading to this type of bond is the same as the reaction leading to the formation of “normal” 3-3 bonds. Double cross-links can be detected in the peptidoglycan from *C. difficile* as well as in the peptidoglycan from Gram-negative organisms producing Ldts (*E. coli*, *Rhizobium leguminosarum*, and *Brucella abortus*; S. Mesnage, unpublished). Interestingly, double 4-3 cross-links resulting from D,D-transpeptidation have also been described in *Staphylococcus aureus* (14). The physiological role of these peptidoglycan cross-links remains unknown and awaits further studies.

Based on the impact of L,D-transpeptidation on antibiotic resistance in *Enterococcus faecium*, it is tempting to assume that L,D-transpeptidation in *C. difficile* could underpin beta-lactam resistance. This remains an open question since we were unable to generate a mutant devoid of 3-3 cross-links. The mutant harboring deletions in the genes encoding the three canonical Ldts still contained 78% of 3-3 cross-links, indicating that this organism encodes (an)other enzyme(s) that does not contain a YkuD domain but is (are) able to make 3-3 cross-links. Our findings are surprising and somewhat contrasting with a previous study, where the combined deletion of *ldt*_*Cd1*_ and *ldt*_*Cd2*_ led to a decrease in the cross-linking index (18.2% as compared with 33.8% for the WT strain). This discrepancy is difficult to explain but could be attributed either to the different strain analyzed (*C. difficile* 630 *versus* R20291) or to the different strategies followed for peptidoglycan analysis. The work by Peltier *et al.* involved offline analysis of individual fractions from a single replicate and a quantification of muropeptides based on UV whilst our analysis involved LC-MS analysis (online) of biological triplicates and a quantification using ion intensity. Based on the remarkably similar peptidoglycan structure of the WT and mutant strain described here (Tables 1 and 2), we are confident that the three canonical Ldt_Cd_ enzymes have a minor contribution to the formation of 3-3 cross-links and only contribute significantly to incorporate noncanonical amino acids in the position 4 of peptide stems.

The interaction of recombinant Ldts with beta-lactams has been extensively studied *in vitro*, and the data reported support the idea that these enzymes play a role in resistance to these antibiotics. Ldts are acylated by beta-lactams, but enzyme inactivation only occurs in the presence of carbapenems and penems. Other beta-lactams such as cephems (cephalosporin) are poor inhibitors since acylation is slow and the thioester bond formed in the enzyme–antibiotic adduct is prone to hydrolysis (15). This has been shown for model organisms including *E. faecium* (15), *Mycobacterium tuberculosis* (16), and *C. difficile* Ldts (8) and is true for most Ldts studied to date despite some exceptions for *M. tuberculosis* Ldt_Mt5_ (16), *Acinetobacter baumanii* Ldt_Ab_ (17), and *C. difficile* Ldt_Cd3_ (8), which are not inhibited by carbapenems. The contribution of Ldts to beta-lactam resistance has been documented in *E. faecium* (18), *M. tuberculosis* (19), *Mycobacterium smegmatis* (20), and *A. baumanii* (17). In *C. difficile*, the inactivation of two of the three Ldts did not lead to a change in beta-lactam resistance (6). Our data revealed that the inactivation of all canonical *C. difficile* ldts has no impact on beta-lactam resistance, as expected, based on the results from peptidoglycan analysis.

The identification of alternative (noncanonical) Ldt(s) encoded by *C. difficile* is therefore required to investigate (i) whether L,D-transpeptidation is essential in this organism and (ii) whether this mode of peptidoglycan polymerization underpins beta-lactam resistance.

## Experimental procedures

### Bacterial strains, plasmids, oligonucleotides, and growth conditions

Bacterial strains, plasmids, and oligonucleotides are described in Table S4. *C. difficile* R20291 (ribotype 027) and isogenic derivatives were grown on BHI agar plates or in TY broth. During selection of mutants, strains were grown on *C. difficile* minimal medium (21) supplemented with 5-fluorocytosine (50 μg/ml) when required. Cultures were incubated at 37 °C in an anaerobic cabinet under an atmosphere containing 80% nitrogen, 10% hydrogen, and 10% carbon dioxide. *E. coli* was grown on Luria Bertani (LB) agar plates or in LB broth at 37 °C. When needed, thiamphenicol was added (30 μg/ml).

## Construction of *C. difficile* deletion mutants

*C. difficile* mutant strains were constructed by homologous recombination. Briefly, 1.2 kb upstream and downstream of the region to be deleted was synthesized as a single DNA fragment (Genewiz) and cloned between BamHI and SacI sites in pJAK112, yielding pNG007 (*ldt*_*Cd1*_ deletion), pNG008 (*ldt*_*Cd2*_ deletion), and pNG009 (*ldt*_*Cd3*_ deletion). Plasmids were introduced into *C. difficile* strain R20291 by conjugation (22), and allelic exchange was carried out as described (10).

## Determination of minimum inhibitory concentrations

MICs were determined according to an agar dilution method using Wilkins Chalgren agar and as recommended by the Clinical and Laboratory Standards Institute guidelines. *C. difficile* isolates were cultured on fresh blood agar plates, prior to inoculation of single colonies into prereduced Schaedler Anaerobic Broths and anaerobic culture for 24 h. Cultures were diluted in prereduced phosphate-buffered saline to achieve a 1 McFarland standard equivalent, and 10^5^ colony-forming units were spotted on Wilkins Chalgren agar containing doubling antibiotic dilutions and non-antibiotic-containing controls. Agar plates containing amoxicillin clavulanate were prepared with a fixed concentration of 2 mg/l clavulanate, and those containing piperacillin tazobactam were prepared with a fixed concentration of 4 mg/l tazobactam, as recommended by European Committee on Antimicrobial Susceptibility Testing guidelines. Agar plates were incubated anaerobically for 48 h before reading. The MIC was defined as the lowest concentration of antibiotic completely preventing growth, significantly reducing it to a haze or one to three discrete colonies.

## Chromosomal DNA extraction, sequencing, and genome analysis

Genomic DNA was purified using phenol–chloroform extraction as described (10), and whole genome sequencing was performed by MicrobesNG using their standard Illumina service. Sequence analysis was performed using a custom script, as described (23). In brief, reads were aligned to the *C. difficile* R20291 reference (accession number: FN545816) using BWA-mem (v0.7.17) and sorted using SAMtools (v1.43) (24). PCR duplicates were removed *via* Picard (v2.25.2) (http://broadinstitute.github.io/picard/). SAMtools (v1.43) mpileup was used to generate the mpileup prerequisite for Varscan. Varscan (v2.4.3-1) (25) was then used to call variants using parameters previously described (23), and snpEff (v5.0) (26) was used to annotate variants. Variants that co-occurred in the WT were removed to generate a list of mutations unique to mutant strains. Mutations were visualized on the genome using a previously published custom script in RStudio (v4.1.0) using the Plotrix package (27).

## TraDIS analysis

The construction of the transposon library, the sequencing of insertion sites, and the mapping to their corresponding reference sequences were described (10). Visualization of insertion sites was done using the Artemis genome browser (28).

## Peptidoglycan extraction

*C. difficile* strains were grown overnight in 10 ml of TY broth from a single colony. The starter cultures were used to inoculate 100 ml TY medium (1/100 dilution). After 48 h at 37 °C, cells were spun, supernatant was discarded, and cell pellet was snap frozen in liquid nitrogen; the cell pellet was then resuspended in 20 ml of boiling MilliQ water (MQ) before the addition of 5 ml warm 20% (w/v) SDS (4% SDS final concentration). After 30 min at 100 °C, the cells were allowed to cool down to room temperature. Insoluble cell walls were pelleted at 45,000*g* for 20 min and washed 5 times using warm MQ water. Proteins covalently bound to peptidoglycan were removed by pronase treatment (final concentration of 2 mg/ml, 4 h at 60 °C). Protease-treated cell walls were washed 6 times with 30 ml of MQ water before covalently bound polymers were removed by incubation in 1 M HCl for 5 h at 37 °C. Insoluble pure peptidoglycan was washed 6 times with MQ water, snap frozen in liquid nitrogen, freeze-dried and resuspended at a final concentration of 10 mg/ml.

## Ldt_Cd_ production and purification

The plasmids for protein production were designed as described (8). Ldt_Cd1_ and Ldt_Cd3_ were expressed as full-length His-tagged proteins. Ldt_Cd2_ could not be produced as a stable full-length protein, so the catalytic domain was purified. Recombinant Ldt_Cd_ were produced in *E. coli* BL21(DE3) grown in LB broth. One-liter cultures were inoculated at an OD_600nm_ of 0.05, and protein expression was induced with 1 mM isopropyl ß-D-1-thiogalactopyranoside when the cultures reached an OD_600nm_ of 0.7. They were then cooled down to 20 °C and incubated for 16 h at this temperature. Cells were harvested, resuspended in a buffer containing 50 mM Tris-HCl (pH8.0) + 500 mM NaCl, and mechanically broken using a French press (2 passages at 1250 psi). Cell debris were removed by centrifuging the crude cell extract at 45,000*g* for 30 min at 4 °C. The entire soluble fraction was loaded on a 5-ml HiTrap column equilibrated in buffer A at a flow rate of 5 ml/min. Elution was performed using a 10 column volume gradient to 250 mM imidazole in buffer A. Fractions containing the Ldt_Cd_ proteins were pooled, concentrated to 2 mg/ml and further purified by gel filtration chromatography using a Hiload 16/600 superdex 75 column equilibrated in 50 mM Tris-HCl pH 8.5 + 250 mM NaCl). Ldt_Cd_ proteins were concentrated on an Amicon centrifugal filter to a final concentration of 2 mg/ml and stored at −80 °C until further use.

## Purification of substrates for *in vitro* assays

Peptidoglycan fragments used as substrates were purified from *E. coli* or *C. difficile* sacculi digested with mutanolysin and reduced with sodium borohydride. Digestion products were separated by reversed-phase HPLC using a Hypersil column (4.6 mm × 250 mm, 5 μm particle size) using a water–acetonitrile–0.1% (v/v) formic acid gradient. Fractions containing the muropeptides of interest were freeze-dried and quantified by NMR using trimethylsilyl propionate as a standard.

## *In vitro* Ldt assays

Each *in vitro* assay was carried out in triplicate, and average chromatograms are shown in Figure 1. Each reaction was carried out in a phosphate saline buffer (pH 8.0) in a final volume of 50 μl and contained 100 μM substrate and 10 μM enzyme. For exchange reactions, D-methionine was added at a concentration of 1 mM. Reactions were incubated at 37 °C for 4 h.

## Preparation of soluble muropeptides for peptidoglycan structural analysis

Purified peptidoglycan (1 mg) was digested overnight in 50 mM phosphate buffer (pH 5.5) supplemented with 200 U of mutanolysin (Sigma) in a final volume of 125 μl. Following heat inactivation of mutanolysin (5 min at 100 °C), soluble disaccharide peptides were mixed with an equal volume of 250 mM borate buffer (pH 9.25) and reduced with 0.2% (w/v) sodium borohydride. After 20 min at room temperature, the pH was adjusted to 4.5 to 5.5 using phosphoric acid.

## Ultrahigh-Performance chromatography coupled to tandem mass spectrometry

An Ultimate 3000 UHPLC (Dionex/Thermo Fisher Scientific) system coupled with a high-resolution Q Exactive Focus mass spectrometer (Thermo Fisher Scientific) was used for LC-MS analysis. Muropeptides were separated using a C18 analytical column (Hypersil Gold aQ, 1.9-μm particles, 150 mm × 2.1 mm; Thermo Fisher Scientific) at a temperature of 50 °C for peptidoglycan analysis or on a smaller C18 column for *in vitro* assays (Hypersil Gold aQ, 1.9-μm particles, 50 mm × 2.1 mm; Thermo Fisher Scientific). For peptidoglycan analysis, muropeptide elution was performed at 0.25 ml/min by applying a mixture of solvent A (water, 0.1% [v/v] formic acid) and solvent B (acetonitrile, 0.1% [v/v] formic acid). Liquid chromatography conditions were 0 to 12.5% B for 25 min increasing to 20% B for 10 min. After 5 min at 95%, the column was re-equilibrated for 10 min with 100% buffer A. For *in vitro* assays, a flow rate of 0.4 ml/min was used. PG fragments were eluted with a 5-min gradient to 15% B followed by 2 min at 95% B. The column was re-equilibrated for 6 min with 100% buffer A.

The Orbitrap Exploris 240 was operated under electrospray ionization (H-ESI high flow)-positive mode, full scan (*m/z* 150–2250) at resolution 120,000 (full width at half maximum) at *m/z* 200, with normalized AGC Target 100%, and automated maximum ion injection time. Data-dependent MS/MS were acquired on a “Top 5” data-dependent mode using the following parameters: resolution 30,000; AGC 100%, automated injection time, with normalized collision energy 25%.

## Nuclear magnetic resonance

Purified peptidoglycan fragments were dissolved in 90% H_2_O/10% D_2_O. They were analyzed by NMR at 298 K on a Bruker DRX-600 equipped with a cryoprobe. TOCSY spectra were acquired using a 60-ms spin-lock with a field strength of 10 kHz. NOESY spectra used a 200-ms mixing time. All data were analyzed using Topspin 4.0.5.

## Analysis of PG structure

LC-MS datasets were deconvoluted with the Byos software v3.11 (Protein Metrics). Sequential searches were carried out with PGFinder v1.0.3, with default settings (10 ppm tolerance, 0.5 min cleanup window) following the strategy described in Fig. S4. Data from individual matching output was consolidated as previously described to calculate average intensities, retention times, observed monoisotopic masses, and ppm differences. The output from individual searches and consolidated data are described in File S2). Cross-linking index and glycan chain length were determined as previously (29). The cross-linking index is defined as 0.5 ∗ (% of dimers) + 0.66 ∗ (% of trimers); glycan chain length was inferred from the abundance of anhydroMurNAc groups, which are found at the end of glycan chains. It is defined as 1/(% of AnhydroMurNAc monomers + 0.5 ∗ (% of AnhydroMurNAc dimers) + 0.33 ∗ (% of AnhydroMurNAc trimers).

## Flow cytometry

Cells corresponding to biological replicates were grown overnight, diluted 1:100 into fresh broth (OD_600_ ∼ 0.02), and grown to mid-exponential phase (OD_600_ ∼ 0.5). Bacteria were diluted 1:100 in filtered phosphate-buffered saline and analyzed by flow cytometry using Millipore Guava Easy Cyte system. Light scatter data were obtained with logarithmic amplifiers for 2500 events. Forward scattered and side-scattered light values were compared using Student *t* test with Welch’s correction using GraphPad Prism.

## Sporulation

Sporulation efficiency was assessed as described (30). Briefly, stationary phase cultures of *C. difficile* were incubated anaerobically for 5 days and the total and heat-resistant (spore) colony-forming units (65 °C for 30 min) were determined every 24 h. Strains were assayed in technical triplicate and the data presented as the mean and standard deviation.

## Toxin release assays

Toxin production was detected in whole cell lysates or concentrated culture supernatants by Western blot. For both fractions, material corresponding to the equivalent of 20 ml of culture at OD_600nm_ = 1 was loaded onto a 6% SDS PAGE, transferred on a polyvinylidene fluoride membrane, and probed with a mouse monoclonal antibody (MA1-7413, Thermo Fisher) against toxin B at a 1/1000 dilution. A secondary rabbit anti-mouse antibody coupled to horseradish peroxidase (#31450, Thermo Fisher) was used at a 1/10,000 dilution. Blots were revealed by chemiluminescence using a BioRad chemidoc system.

## Data availability

LC-MS/MS datasets have been deposited in the GLYCOPOST repository (GPST000371). NMR assignments have been deposited in the Biological Magnetic Resonance Data Bank (52169). Sequencing data were deposited in the NCBI Sequence Read Archive (SRA) under Bioproject ID PRJNA1026070.

## Supporting information

This article contains supporting information (Figs. S1–S8, Tables S1–S4 and Files S1 and S2).

## Conflict of interest

The authors declare that they have no conflicts of interest with the contents of this article.

## References

[1] Wingen-Heimann S.M., Davies K., Viprey V.F., Davis G., Wilcox M.H., Vehreschild M. (2023). *Clostridioides difficile* infection (CDI): a pan-European multi-center cost and resource utilization study, results from the Combatting Bacterial Resistance in Europe CDI (COMBACTE-CDI). Clin. Microbiol. Infect..

[2] Lessa F.C., Gould C.V., McDonald L.C. (2012). Current status of *Clostridium difficile* infection epidemiology. Clin. Infect. Dis..

[3] Buddle J.E., Fagan R.P. (2023). Pathogenicity and virulence of *Clostridioides difficile*. Virulence.

[4] Frere J.M. (1977). Mechanism of action of beta-lactam antibiotics at the molecular level. Biochem. Pharmacol..

[5] Cho H., Uehara T., Bernhardt T.G. (2014). Beta-lactam antibiotics induce a lethal malfunctioning of the bacterial cell wall synthesis machinery. Cell.

[6] Peltier J., Courtin P., El Meouche I., Lemee L., Chapot-Chartier M.P., Pons J.L. (2011). *Clostridium difficile* has an original peptidoglycan structure with a high level of N-acetylglucosamine deacetylation and mainly 3-3 cross-links. J. Biol. Chem..

[7] Aliashkevich A., Cava F. (2021). L,D-transpeptidases: the great unknown among the peptidoglycan cross-linkers. FEBS J..

[8] Sutterlin L., Edoo Z., Hugonnet J.E., Mainardi J.L., Arthur M. (2018). Peptidoglycan cross-linking activity of L,D-Transpeptidases from *Clostridium difficile* and inactivation of these enzymes by beta-lactams. Antimicrob. Agents Chemother..

[9] More N., Martorana A.M., Biboy J., Otten C., Winkle M., Serrano C.K.G. (2019). Peptidoglycan remodeling enables *Escherichia coli* to survive severe outer membrane assembly defect. mBio.

[10] Dembek M., Barquist L., Boinett C.J., Cain A.K., Mayho M., Lawley T.D. (2015). High-throughput analysis of gene essentiality and sporulation in *Clostridium difficile*. mBio.

[11] Patel A.V., Turner R.D., Rifflet A., Acosta-Martin A.E., Nichols A., Awad M.M. (2021). PGFinder, a novel analysis pipeline for the consistent, reproducible and high-resolution structural analysis of bacterial peptidoglycans. eLife.

[12] Voedts H., Dorchene D., Lodge A., Vollmer W., Arthur M., Hugonnet J.E. (2021). Role of endopeptidases in peptidoglycan synthesis mediated by alternative cross-linking enzymes in Escherichia coli. EMBO J..

[13] Chodisetti P.K., Reddy M. (2019). Peptidoglycan hydrolase of an unusual cross-link cleavage specificity contributes to bacterial cell wall synthesis. Proc. Natl. Acad. Sci. U. S. A..

[14] Boneca I.G., Xu N., Gage D.A., de Jonge B.L., Tomasz A. (1997). Structural characterization of an abnormally cross-linked muropeptide dimer that is accumulated in the peptidoglycan of methicillin- and cefotaxime-resistant mutants of *Staphylococcus aureus*. J. Biol. Chem..

[15] Triboulet S., Dubee V., Lecoq L., Bougault C., Mainardi J.L., Rice L.B. (2013). Kinetic features of L,D-transpeptidase inactivation critical for beta-lactam antibacterial activity. PLoS One.

[16] Cordillot M., Dubee V., Triboulet S., Dubost L., Marie A., Hugonnet J.E. (2013). *In vitro* cross-linking of Mycobacterium tuberculosis peptidoglycan by L,D-transpeptidases and inactivation of these enzymes by carbapenems. Antimicrob. Agents Chemother..

[17] Toth M., Stewart N.K., Smith C.A., Lee M., Vakulenko S.B. (2022). The l,d-transpeptidase ldt(Ab) from *Acinetobacter baumannii* is poorly inhibited by carbapenems and has a unique structural architecture. ACS Infect. Dis..

[18] Mainardi J.L., Legrand R., Arthur M., Schoot B., van Heijenoort J., Gutmann L. (2000). Novel mechanism of beta-lactam resistance due to bypass of DD-transpeptidation in *Enterococcus faecium*. J. Biol. Chem..

[19] Gupta R., Lavollay M., Mainardi J.L., Arthur M., Bishai W.R., Lamichhane G. (2010). The *Mycobacterium tuberculosis* protein LdtMt2 is a nonclassical transpeptidase required for virulence and resistance to amoxicillin. Nat. Med..

[20] Baranowski C., Welsh M.A., Sham L.T., Eskandarian H.A., Lim H.C., Kieser K.J. (2018). Maturing *Mycobacterium smegmatis* peptidoglycan requires non-canonical crosslinks to maintain shape. eLife.

[21] Karasawa T., Ikoma S., Yamakawa K., Nakamura S. (1995). A defined growth medium for *Clostridium difficile*. Microbiology (Reading).

[22] Kirk J.A., Fagan R.P. (2016). Heat shock increases conjugation efficiency in *Clostridium difficile*. Anaerobe.

[23] Buddle J.E., Wright R.C.T., Turner C.E., Chaudhuri R.R., Brockhurst M.A., Fagan R.P. (2023). Multiple evolutionary pathways lead to vancomycin resistance in *Clostridioides difficile*. bioRxiv.

[24] Li H., Durbin R. (2009). Fast and accurate short read alignment with Burrows-Wheeler transform. Bioinformatics.

[25] Koboldt D.C., Chen K., Wylie T., Larson D.E., McLellan M.D., Mardis E.R. (2009). VarScan: variant detection in massively parallel sequencing of individual and pooled samples. Bioinformatics.

[26] Cingolani P., Platts A., Wang le L., Coon M., Nguyen T., Wang L. (2012). A program for annotating and predicting the effects of single nucleotide polymorphisms, SnpEff: SNPs in the genome of Drosophila melanogaster strain w1118; iso-2; iso-3. Fly (Austin).

[27] Harrison E., Guymer D., Spiers A.J., Paterson S., Brockhurst M.A. (2015). Parallel compensatory evolution stabilizes plasmids across the parasitism-mutualism continuum. Curr. Biol..

[28] Carver T., Harris S.R., Berriman M., Parkhill J., McQuillan J.A. (2012). Artemis: an integrated platform for visualization and analysis of high-throughput sequence-based experimental data. Bioinformatics.

[29] Glauner B. (1988). Separation and quantification of muropeptides with high-performance liquid chromatography. Anal. Biochem..

[30] Kirk J.A., Gebhart D., Buckley A.M., Lok S., Scholl D., Douce G.R. (2017). New class of precision antimicrobials redefines role of *Clostridium difficile* S-layer in virulence and viability. Sci. Transl. Med..

