# Supplementary material for: Clostridioides difficile canonical L,D-transpeptidases catalyze a novel type of peptidoglycan cross-links and are not required for beta-lactam resistance
Source: J Biol Chem. 2023 Dec 1;300(1):105529. doi: 10.1016/j.jbc.2023.105529 (PMC10792238; doi:10.1016/j.jbc.2023.105529)
Supplement: Supplementary Figures S1 to S8 [file mmc3.pdf]

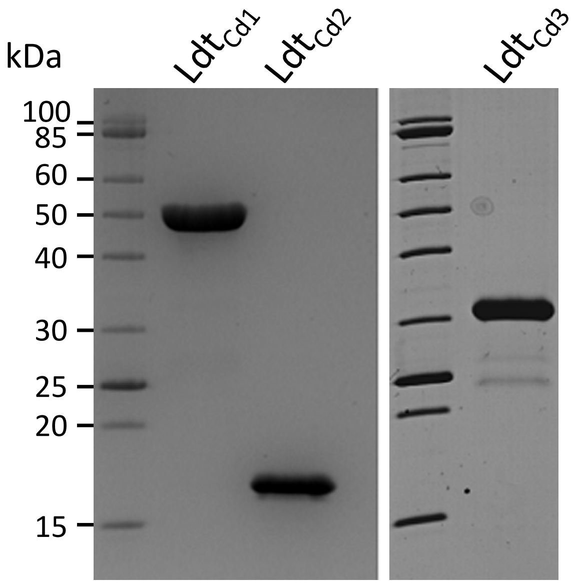

Figure S1. SDS-PAGE analysis of Ldt$_{Cd1}$, Ldt$_{Cd2}$ and Ldt$_{Cd3}$ purified recombinant proteins used for *in vitro* assays.

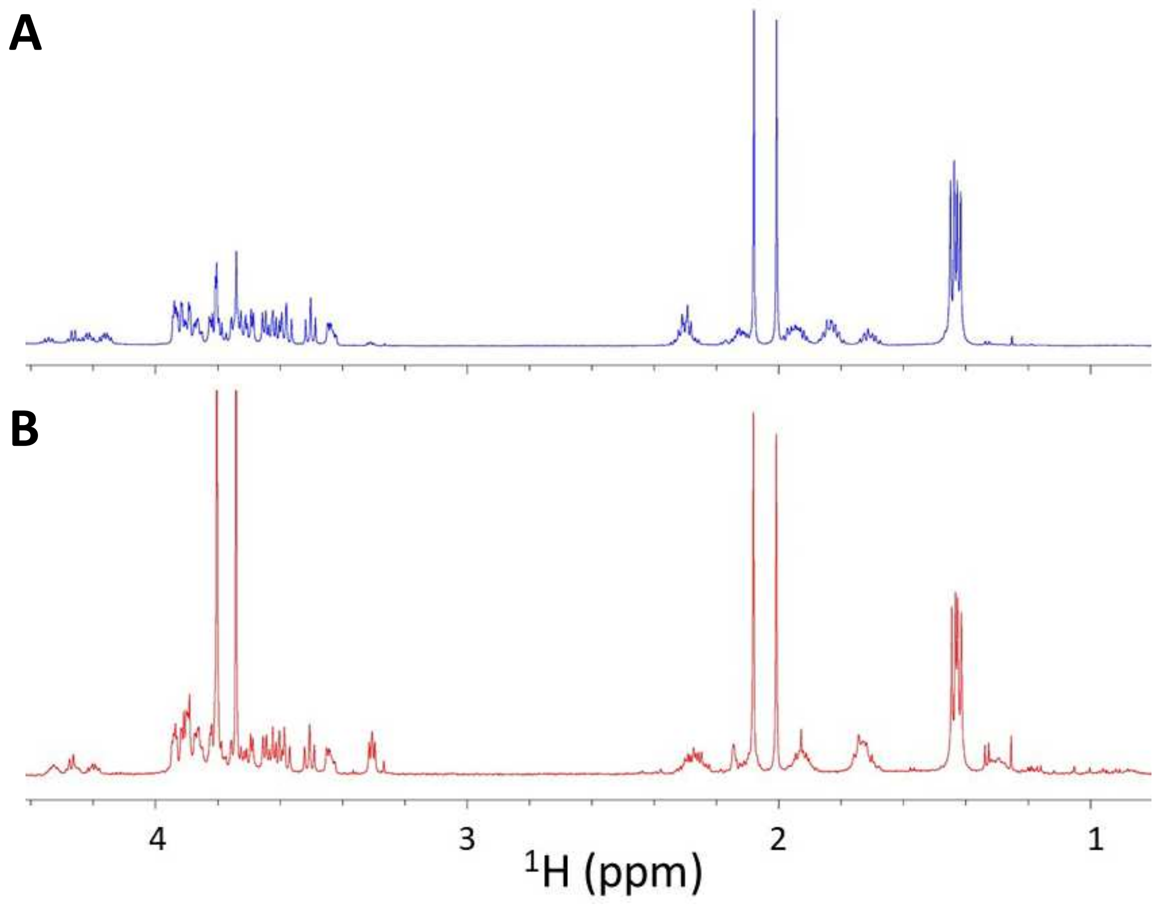

Figure S2. One-dimensional ^{1}H NMR spectra of (**A**) gm-AEJ monomer (peak 1 in Figure 1) and (**B**) gm-AEJ=gm-AEJ (-H2O) dimer (peak a in Figure 1). The two singlet *N*-acetyl methyl signals are at around 2 ppm, and the two methyl doublets are at around 1.4 ppm.

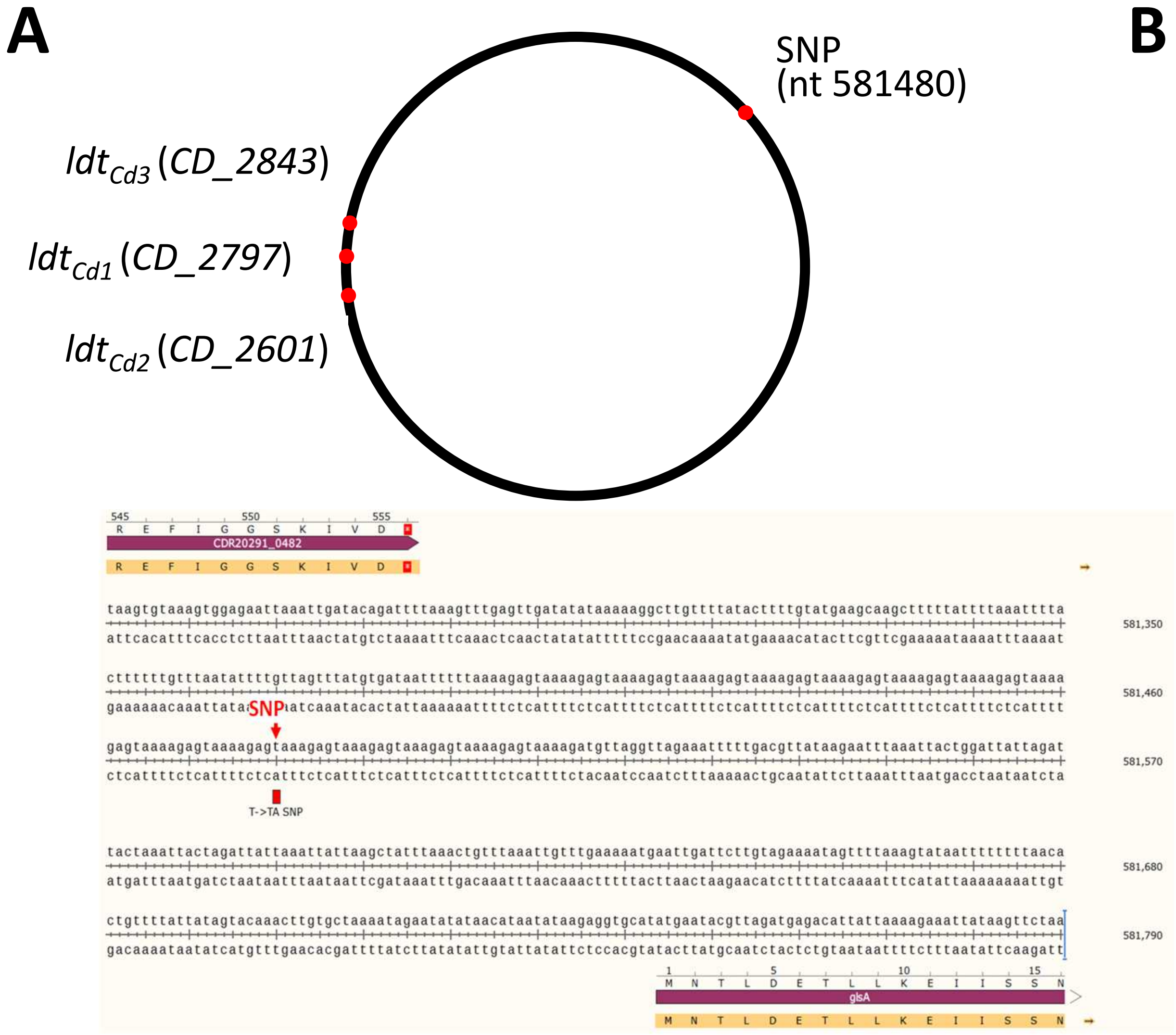

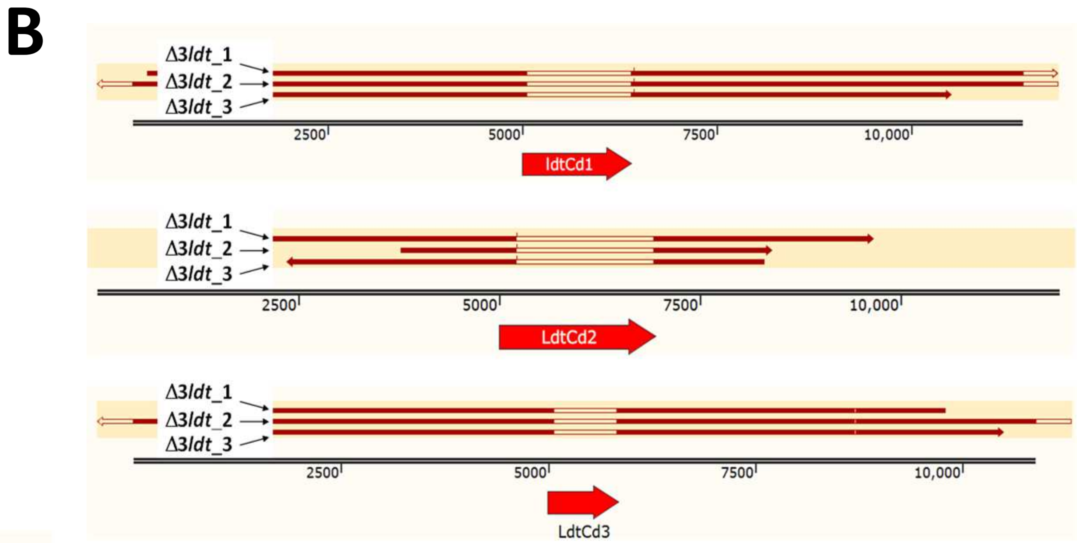

Figure S3. Genome analysis of *C. difficile* WT and Δ3*ldt* derivative. **A**, schematic representation of the R20291 chromosome depicting the localisation of the 3 ldt_{Cd} genes and the SNP detected in the triple mutant in position 581480 (T→TA). The sequence in figure A shows the position of the mutation between the two adjacent genes. **B**, sequence alignments of the contigs assembled from the 3 independent genomes sequenced. Each DNA was purified from the culture that was used for peptidoglycan analysis.

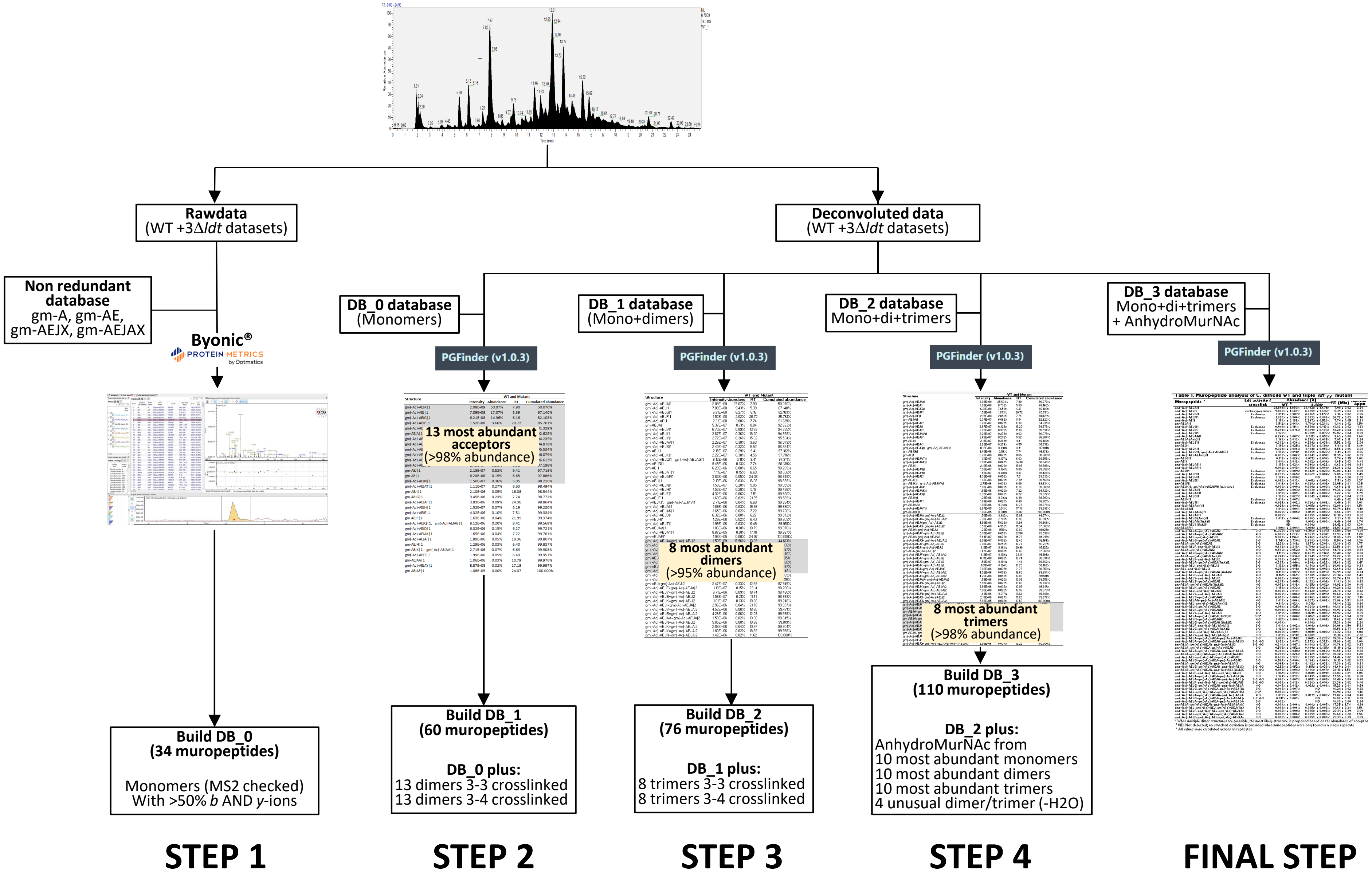

Figure S4. Strategy for peptidoglycan structural analysis. Sequential searches were performed using the Byonic™ and PGFinder software. The monomer database DB_0 was built based on the MS/MS analysis carried out with Byonic™. The identification of the most abundant monomers was used to build the following databases containing dimers (DB_1), trimers (DB_2) and anhydroMurNAc groups (DB_3). DB_3 was used for a final « one off » search.

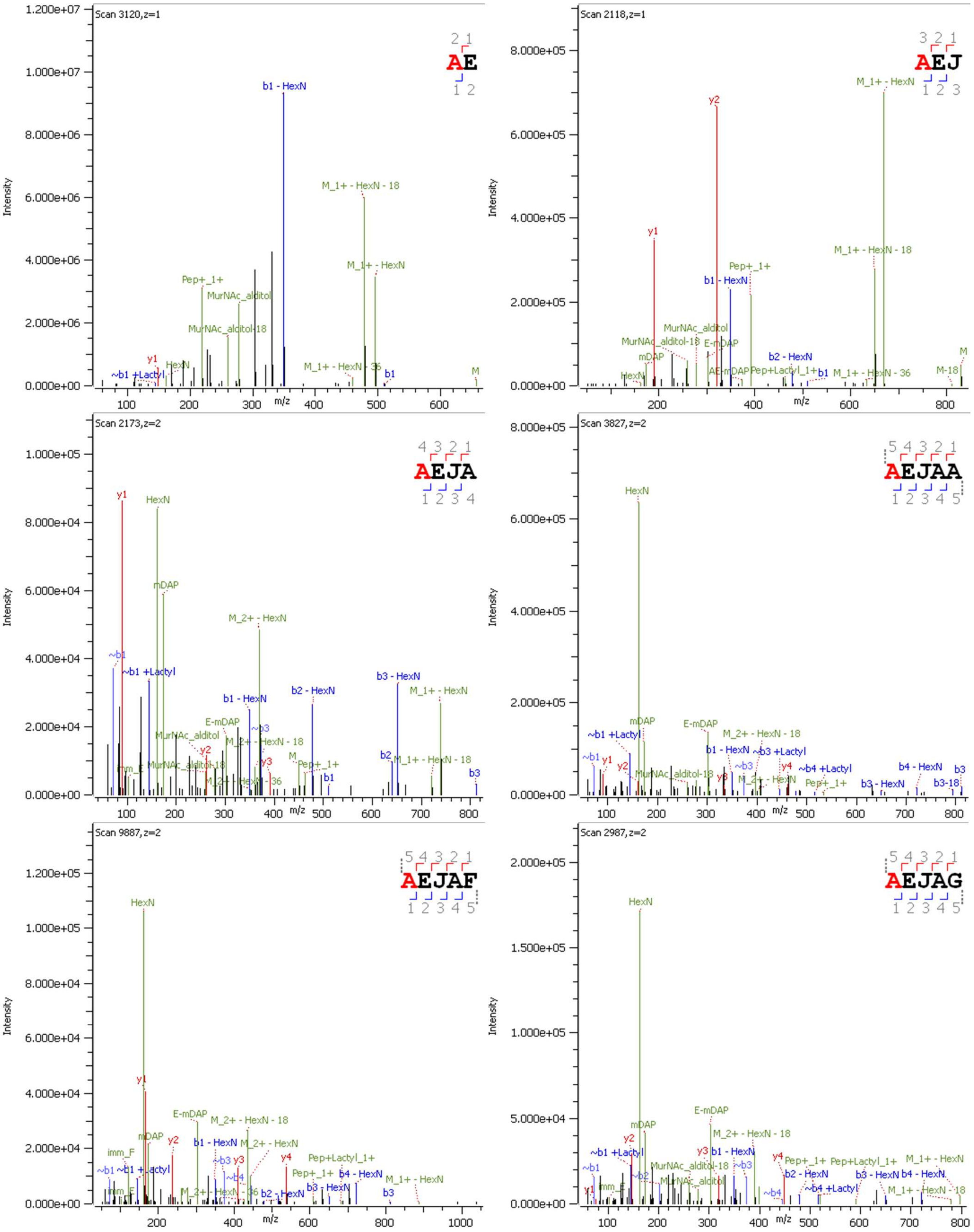
Scan 3120,z=1
AE
b1 - HexN
M_1+ - HexN - 18
M_1+ - HexN
Pep+_1+
MurNAc_alditol
MurNAc_alditol-18
y1
HexN
~b1 +Lactyl
M_1+ - HexN - 36
b1
M
Scan 2118,z=1
AEJ
M_1+ - HexN
y2
y1
M_1+ - HexN - 18
Pep+_1+
b1 - HexN
MurNAc_alditol
MurNAc_alditol-18
E-mDAP
mDAP
b2 - HexN
HexN
AE-mDAP
Pep+Lactyl_1+
M_1+ - HexN - 36
M-18
M
Scan 2173,z=2
AEJA
y1
HexN
mDAP
M_2+ - HexN
~b1
~b1 +Lactyl
b1 - HexN
b2 - HexN
b3 - HexN
M_1+ - HexN
E-mDAP
MurNAc_alditol
M_2+ - HexN - 18
M_1+ - HexN - 18
imm_E
MurNAc_alditol-18
y2
y3
Pep+_1+
b1
b2
b3
M_2+ - HexN - 36
Scan 3827,z=2
AEJAA
HexN
mDAP
E-mDAP
M_2+ - HexN - 18
~b1 +Lactyl
b1 - HexN
~b3 +Lactyl
~b1
y1
y2
~b3
~b4 +Lactyl
b4 - HexN
MurNAc_alditol-18
M_2+ - HexN
Pep+_1+
b3 - HexN
b3
b3-18
Scan 9887,z=2
AEJAF
HexN
y1
E-mDAP
M_2+ - HexN - 18
mDAP
y2
b1 - HexN
M_2+ - HexN
imm_F
~b1
~b1 +Lactyl
~b3
y3
y4
~b4
Pep+Lactyl_1+
Pep+_1+
b4 - HexN
M_1+ - HexN
b3 - HexN
b2 - HexN
b3
M_2+ - HexN - 36
imm_E
Scan 2987,z=2
AEJAG
HexN
E-mDAP
mDAP
M_2+ - HexN - 18
y2
y3
b1 - HexN
~b1 +Lactyl
y4
Pep+_1+
Pep+Lactyl_1+
M_1+ - HexN
MurNAc_alditol-18
~b3
~b1
~b2
b2 - HexN
b3 - HexN
b4 - HexN
MurNAc_alditol
~b4
~b4 +Lactyl
M_1+ - HexN - 18
y1
imm_E
Intensity
m/z

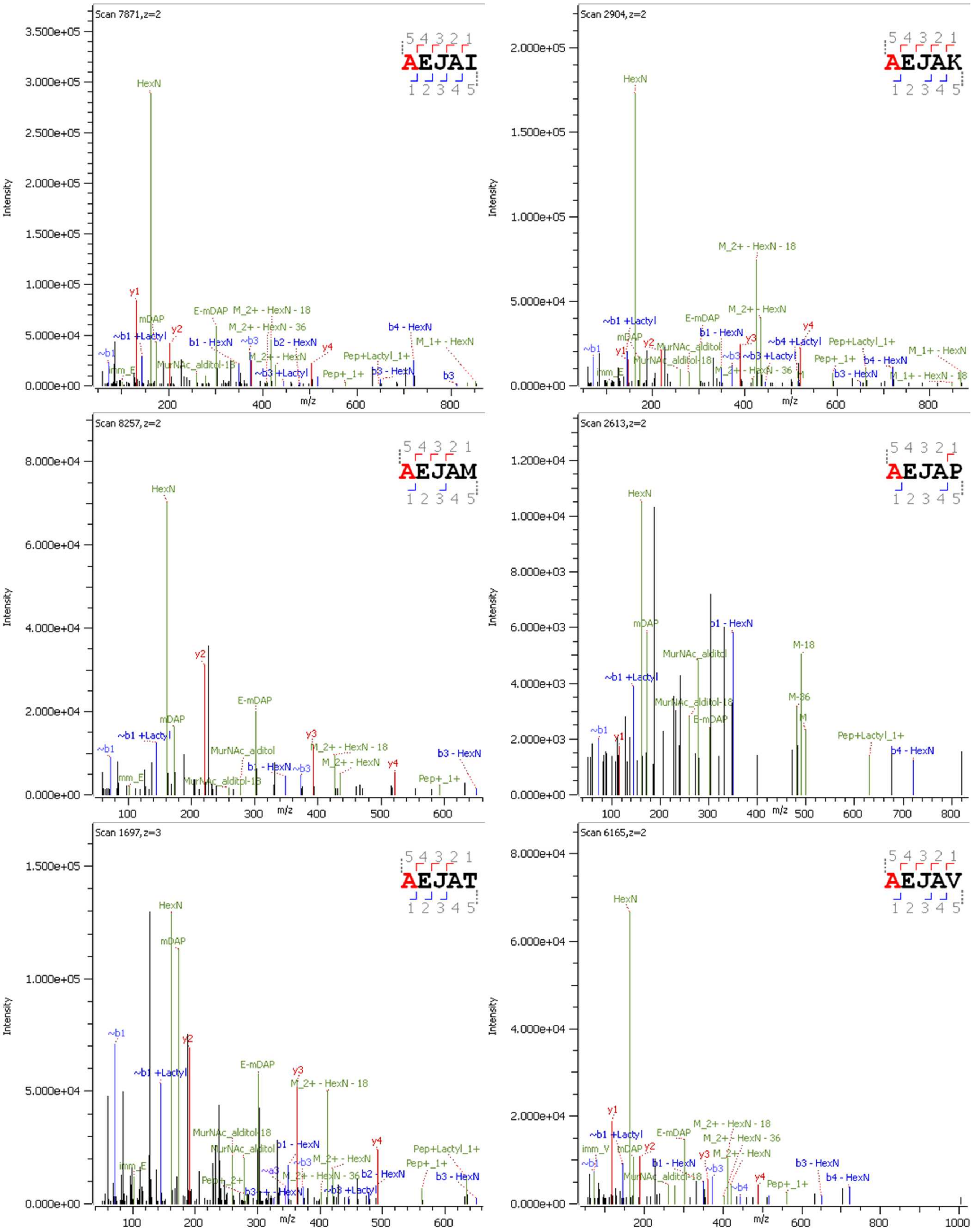

Scan 7871,z=2
AEJAI
HexN
y1
mDAP
~b1 +Lactyl
~b1
imm_E
y2
E-mDAP
M_2+ - HexN - 18
M_2+ - HexN - 36
b1 - HexN
~b3
b2 - HexN
M_2+ - HexN
MurNAc_alditol-18
y4
~b3 +Lactyl
Pep+_1+
Pep+Lactyl_1+
b3 - HexN
b4 - HexN
M_1+ - HexN
b3
Scan 2904,z=2
AEJAK
HexN
M_2+ - HexN - 18
M_2+ - HexN
E-mDAP
~b1 +Lactyl
b1 - HexN
y4
mDAP
~b4 +Lactyl
Pep+Lactyl_1+
~b1
y1
y2
MurNAc_alditol
y3
~b3
~b3 +Lactyl
Pep+_1+
b4 - HexN
M_1+ - HexN
imm_E
MurNAc_alditol-18
M_2+ - HexN - 36
M
b3 - HexN
M_1+ - HexN - 18
Scan 8257,z=2
AEJAM
HexN
y2
E-mDAP
mDAP
~b1 +Lactyl
~b1
y3
M_2+ - HexN - 18
MurNAc_alditol
b3 - HexN
b1 - HexN
~b3
M_2+ - HexN
y4
imm_E
MurNAc_alditol-18
Pep+_1+
Scan 2613,z=2
AEJAP
HexN
mDAP
b1 - HexN
MurNAc_alditol
M-18
~b1 +Lactyl
MurNAc_alditol-18
M-36
E-mDAP
M
~b1
y1
Pep+Lactyl_1+
b4 - HexN
Scan 1697,z=3
AEJAT
HexN
mDAP
~b1
y2
~b1 +Lactyl
E-mDAP
y3
M_2+ - HexN - 18
MurNAc_alditol-18
MurNAc_alditol
b1 - HexN
~b3
M_2+ - HexN
y4
Pep+Lactyl_1+
Pep+_1+
imm_E
~a3
M_2+ - HexN - 36
b2 - HexN
b3 - HexN
Pep+_2+
b3 - HexN
~b3 +Lactyl
Scan 6165,z=2
AEJAV
HexN
y1
M_2+ - HexN - 18
E-mDAP
~b1 +Lactyl
M_2+ - HexN - 36
imm_V
mDAP
y2
y3
M_2+ - HexN
~b1
b1 - HexN
~b3
b3 - HexN
MurNAc_alditol-18
b4 - HexN
y4
~b4
Pep+_1+
Intensity
m/z

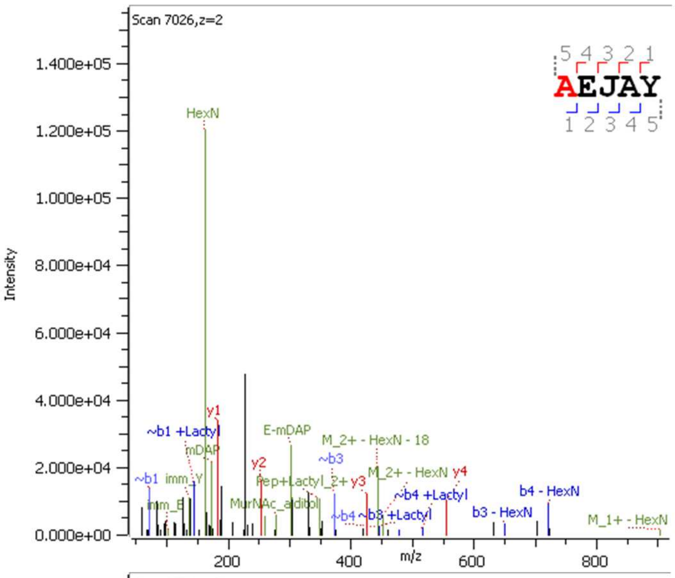
Scan 7026,z=2
AEJAY
Intensity
m/z

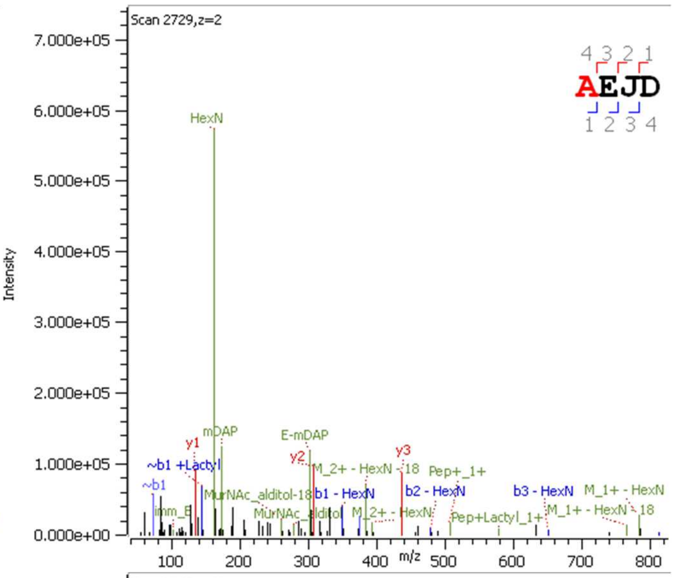
Scan 2729,z=2
AEJD
Intensity
m/z

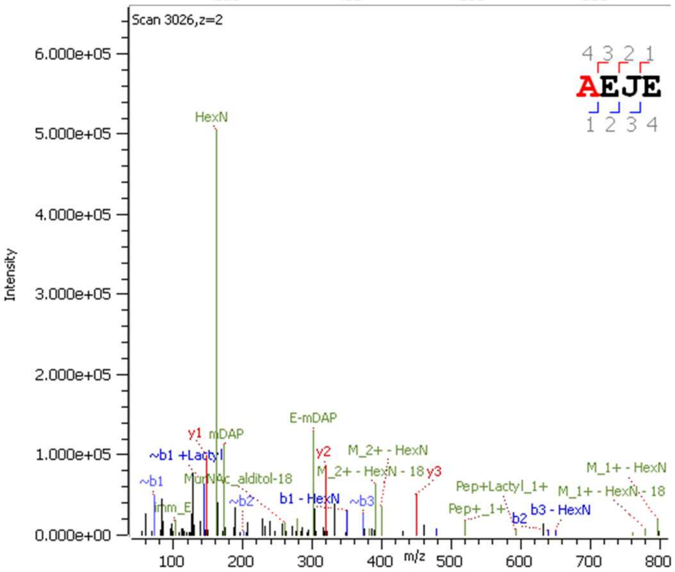
Scan 3026,z=2
AEJE
Intensity
m/z

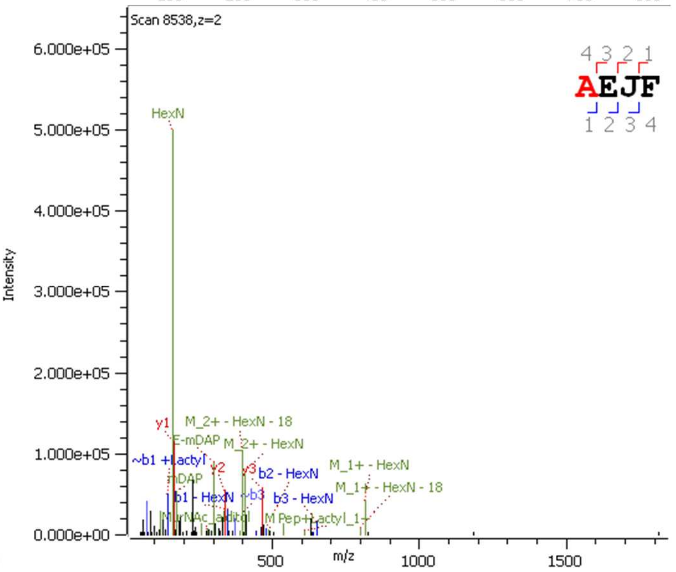
Scan 8538,z=2
AEJF
Intensity
m/z

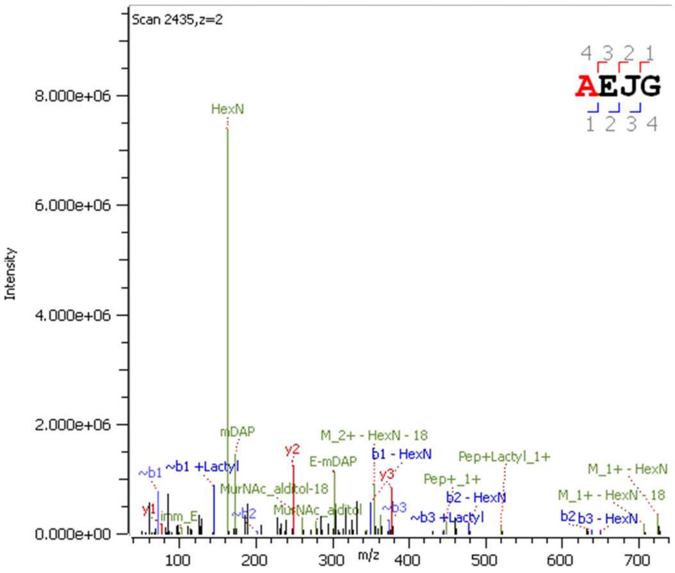
Scan 2435,z=2
AEJG
Intensity
m/z

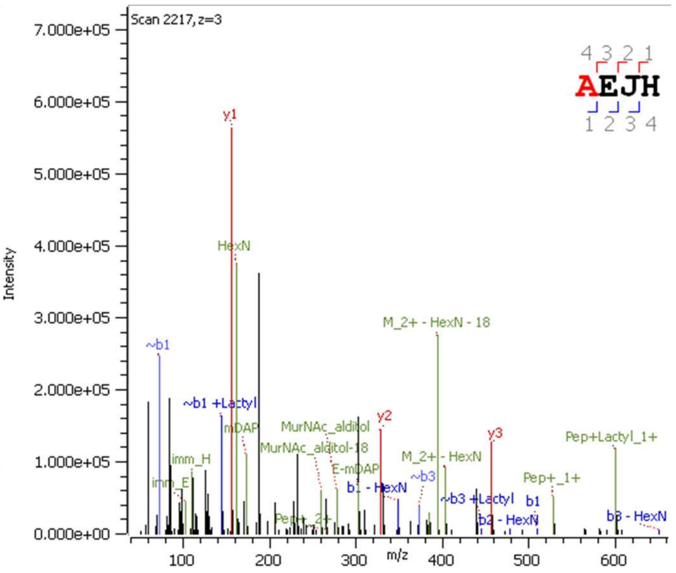
Scan 2217,z=3
AEJH
Intensity
m/z

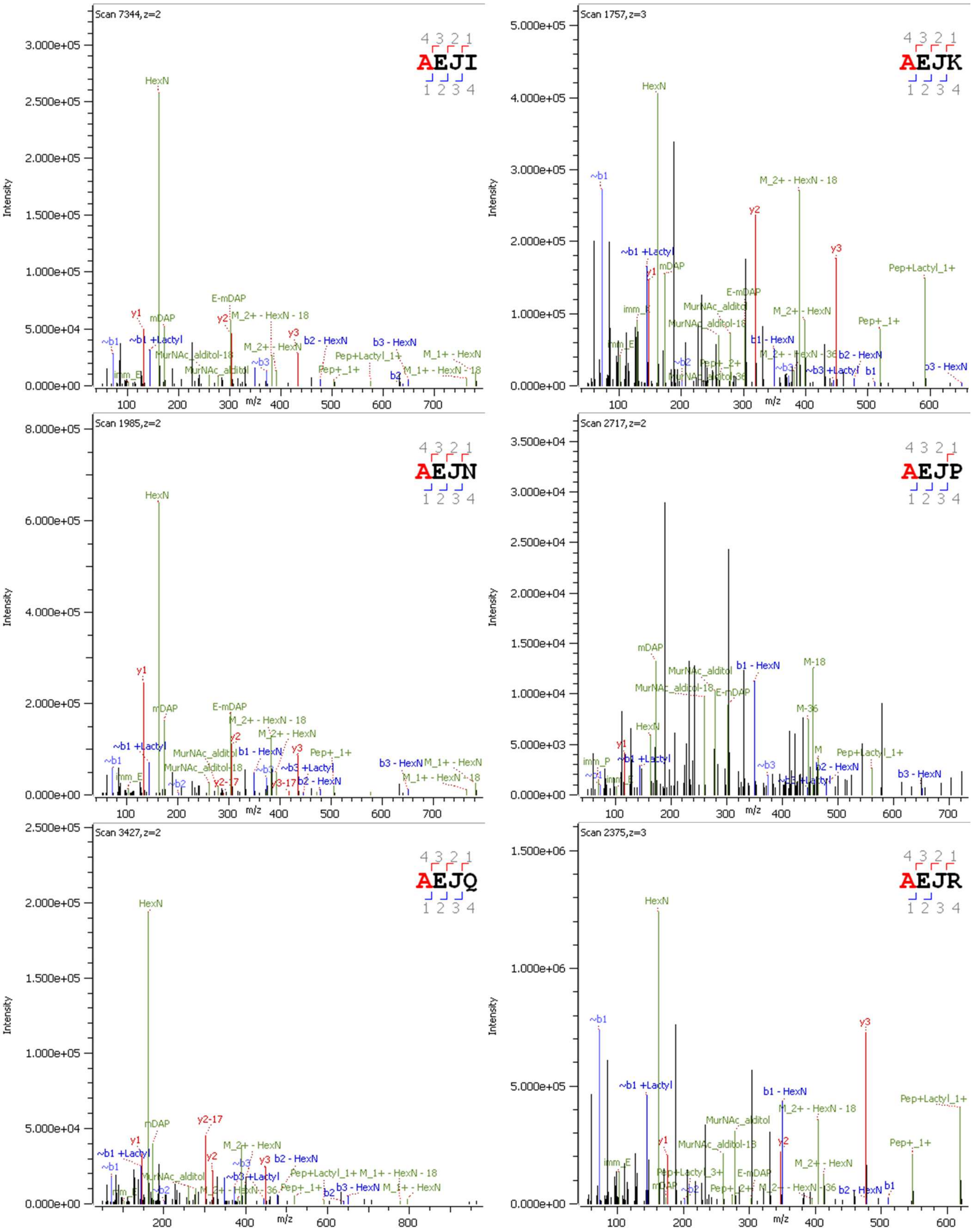
Scan 7344, z=2
AEJI
Scan 1757, z=3
AEJK
Scan 1985, z=2
AEJN
Scan 2717, z=2
AEJP
Scan 3427, z=2
AEJQ
Scan 2375, z=3
AEJR
Intensity
m/z
HexN
mDAP
E-mDAP
M_2+ - HexN - 18
M_2+ - HexN
MurNAc_alditol
MurNAc_alditol-18
Pep+_1+
Pep+Lactyl_1+
M_1+ - HexN
M_1+ - HexN - 18
~b1 +Lactyl
b1 - HexN
b2 - HexN
b3 - HexN
imm_E

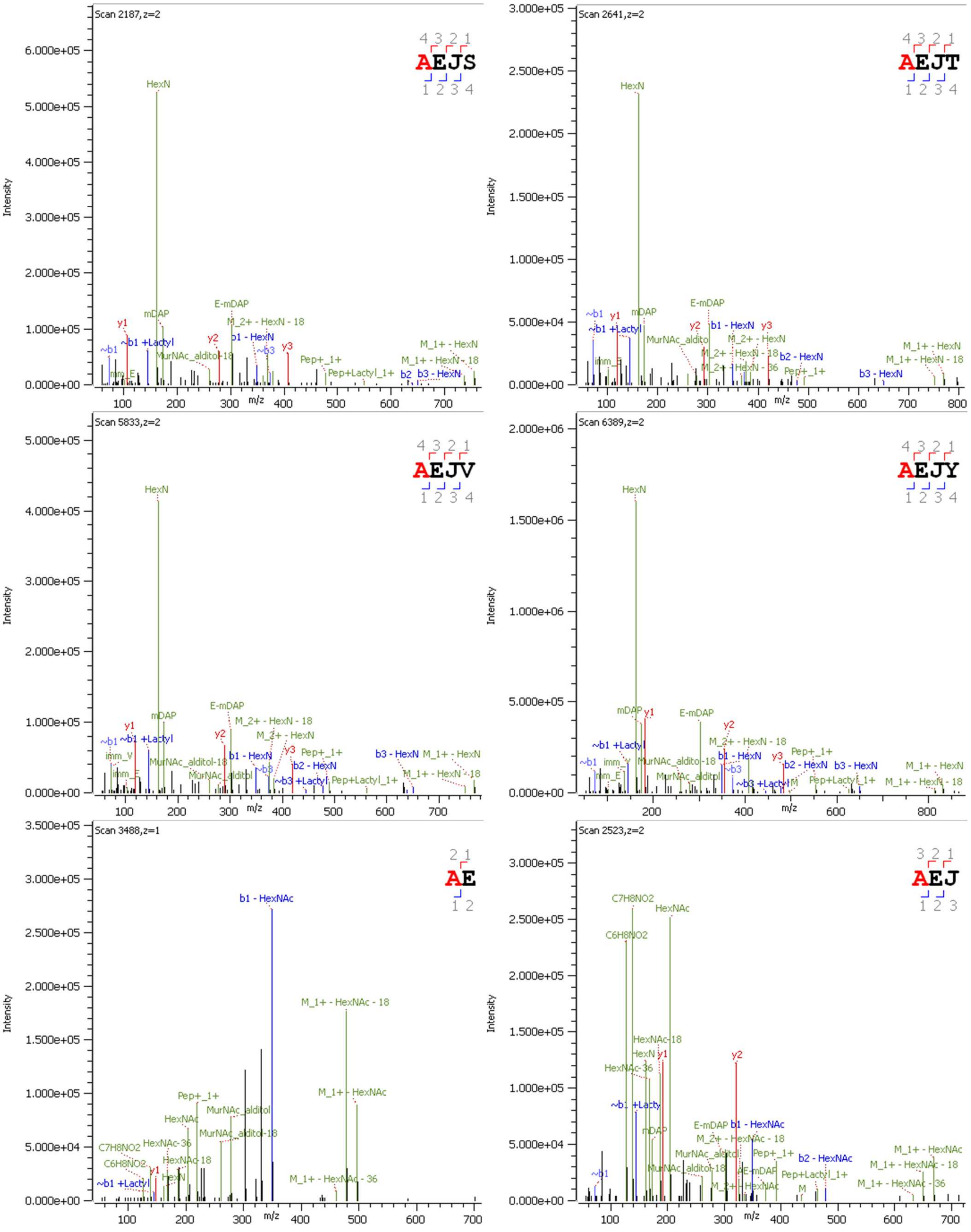

Scan 2187,z=2
AEJS
Intensity
m/z
HexN
mDAP
E-mDAP
M_2+ - HexN - 18
y1
~b1 +Lactyl
y2
b1 - HexN
~b3
y3
~b1
MurNAc_alditol-18
Pep+_1+
imm_E
Pep+Lactyl_1+
b2
b3 - HexN
M_1+ - HexN
M_1+ - HexN - 18
Scan 2641,z=2
AEJT
HexN
E-mDAP
mDAP
~b1
y1
~b1 +Lactyl
y2
b1 - HexN
y3
MurNAc_alditol
M_2+ - HexN
M_2+ - HexN - 18
M_2+ - HexN - 36
b2 - HexN
imm_E
Pep+_1+
b3 - HexN
M_1+ - HexN
M_1+ - HexN - 18
Scan 5833,z=2
AEJV
HexN
E-mDAP
mDAP
M_2+ - HexN - 18
M_2+ - HexN
y1
~b1
~b1 +Lactyl
y2
imm_V
MurNAc_alditol-18
b1 - HexN
y3
Pep+_1+
b3 - HexN
M_1+ - HexN
imm_E
MurNAc_alditol
~b3
b2 - HexN
~b3 +Lactyl
Pep+Lactyl_1+
M_1+ - HexN - 18
Scan 6389,z=2
AEJY
HexN
mDAP
y1
E-mDAP
y2
M_2+ - HexN - 18
~b1 +Lactyl
b1 - HexN
y3
Pep+_1+
~b1
imm_Y
MurNAc_alditol-18
~b3
b2 - HexN
b3 - HexN
M_1+ - HexN
imm_E
MurNAc_alditol
~b3 +Lactyl
M
Pep+Lactyl_1+
M_1+ - HexN - 18
Scan 3488,z=1
AE
b1 - HexNAc
M_1+ - HexNAc - 18
M_1+ - HexNAc
Pep+_1+
MurNAc_alditol
HexNAc
MurNAc_alditol-18
C7H8NO2
HexNAc-36
C6H8NO2
HexNAc-18
y1
HexN
~b1 +Lactyl
M_1+ - HexNAc - 36
Scan 2523,z=2
AEJ
C7H8NO2
HexNAc
C6H8NO2
HexNAc-18
HexN
y1
y2
HexNAc-36
~b1 +Lactyl
mDAP
E-mDAP
b1 - HexNAc
M_2+ - HexNAc - 18
MurNAc_alditol
Pep+_1+
b2 - HexNAc
M_1+ - HexNAc
M_1+ - HexNAc - 18
~b1
MurNAc_alditol-18
AE-mDAP
Pep+Lactyl_1+
M_1+ - HexNAc - 36
M_2+ - HexNAc
M

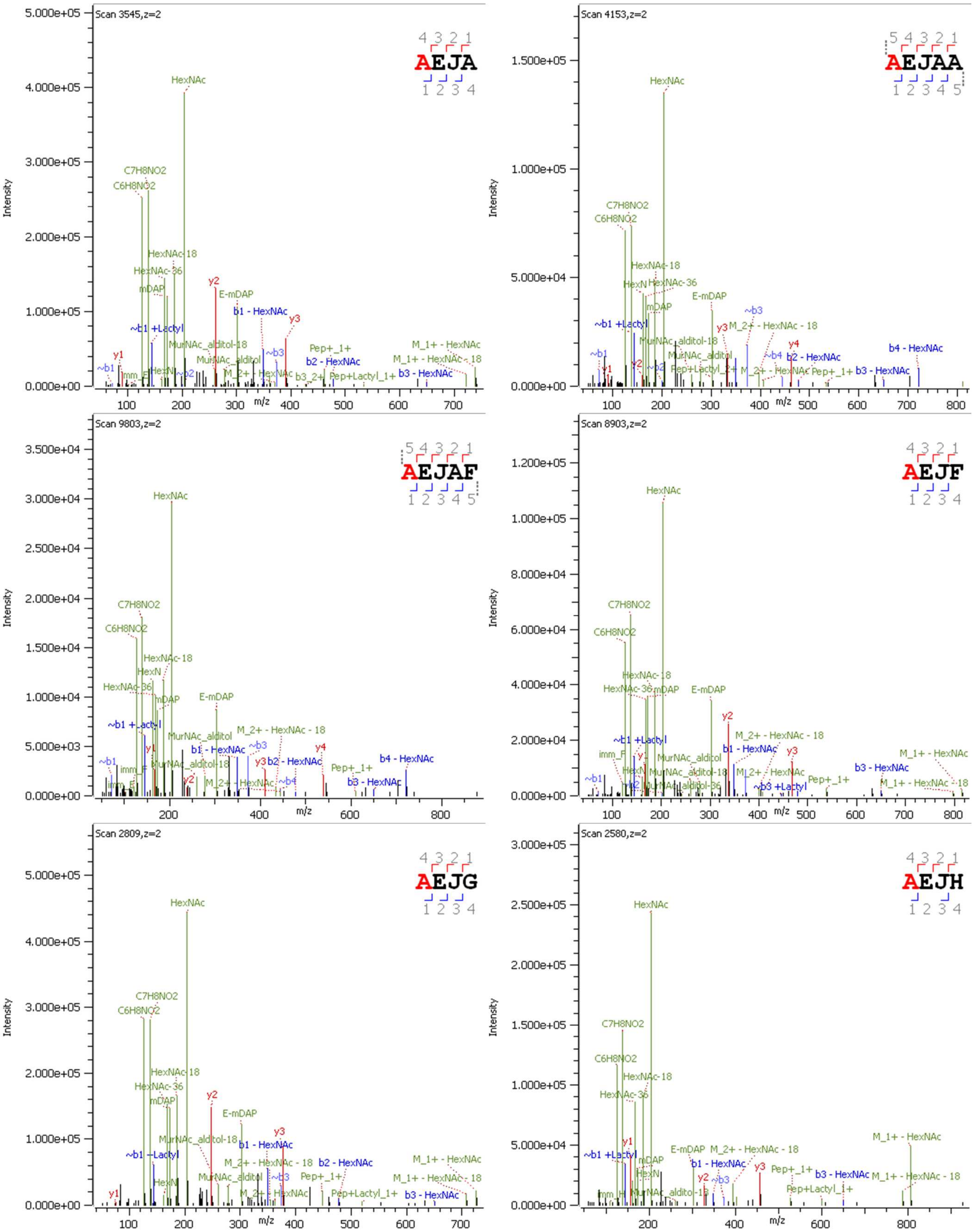
Scan 3545,z=2
AEJA
Scan 4153,z=2
AEJAA
Scan 9803,z=2
AEJAF
Scan 8903,z=2
AEJF
Scan 2809,z=2
AEJG
Scan 2580,z=2
AEJH
Intensity
m/z
HexNAc
C7H8NO2
C6H8NO2
HexNAc-18
HexNAc-36
mDAP
E-mDAP
M_1+ - HexNAc
M_1+ - HexNAc - 18
M_2+ - HexNAc - 18

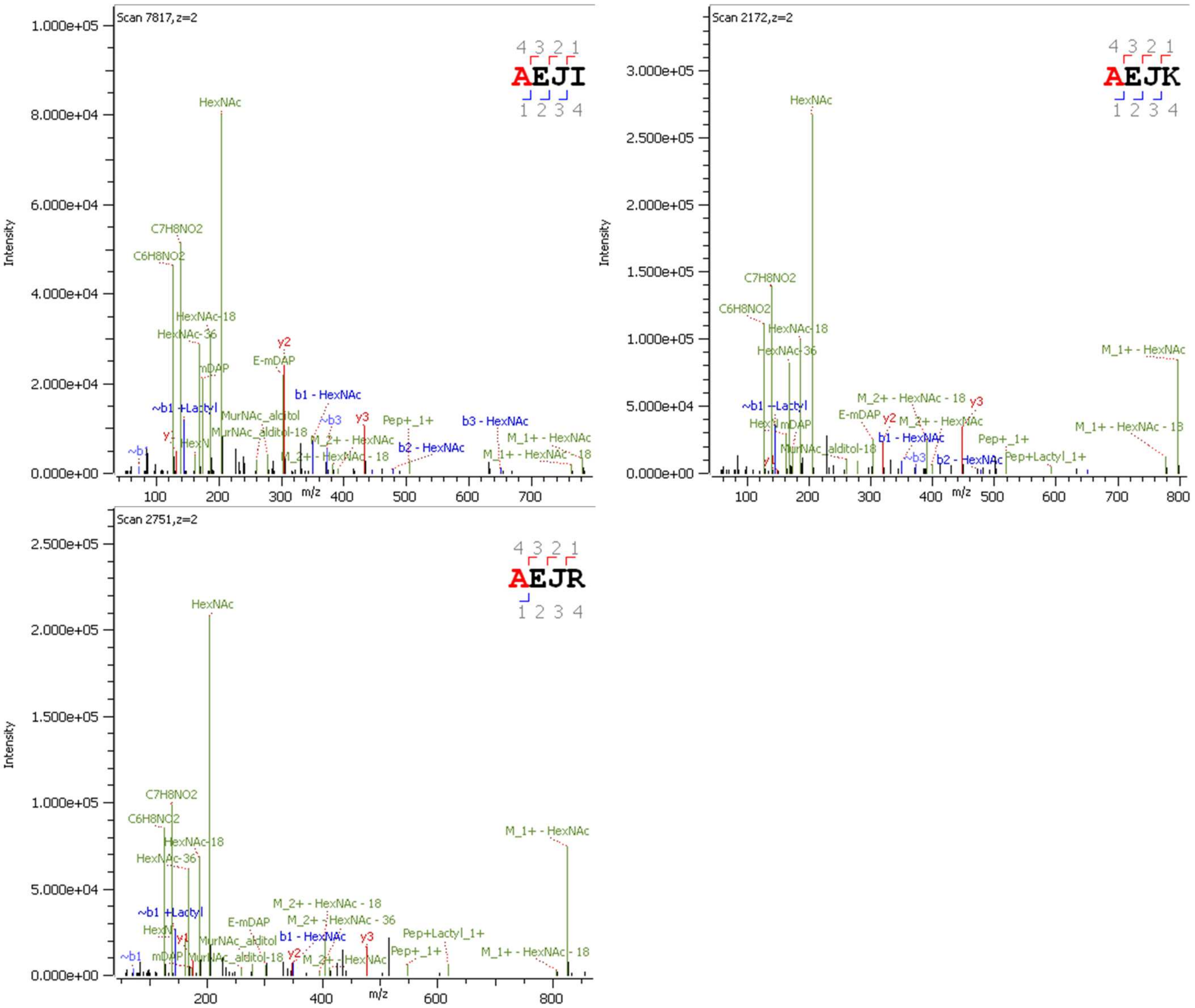

Figure S5. Tandem mass spectrometry analysis of *C. difficile* monomers using the Byonic™ module.

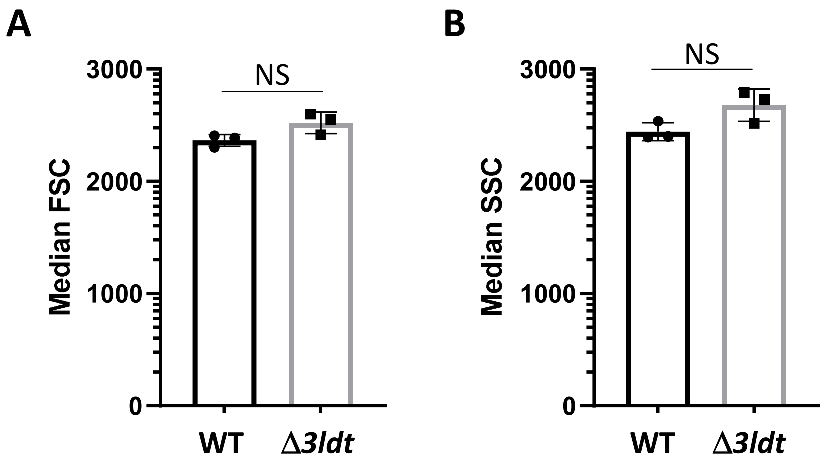

Figure S6. Comparative analysis of WT and Δ*3ldt* cell size by flow cytometry. Comparison of median forward scattered (FSC) (**A**) and side scattered (SSC) light values (**B**) corresponding to WT and Δ3*ldt* mutant; NS, *P*>0.05; n = 3 (Student unpaired *t*-test with Welch's correction).

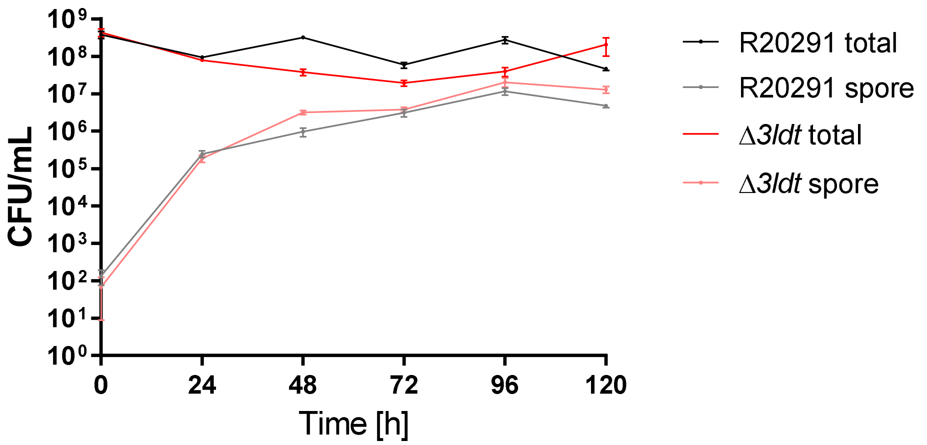

Figure S7. Comparative analysis of WT and Δ3*ldt* sporulation efficiency. Stationary-phase cultures were incubated anaerobically for 5 days, and samples were taken at 24-h intervals for analysis. Total cell numbers were determined by counting the number of CFU on BHIS agar containing the germinant taurocholate. Spore numbers were determined by the same method following incubation at 70°C for 30 min. Experiments were carried out in triplicate on biological duplicate samples. The means ± standard deviations (error bars) are shown.

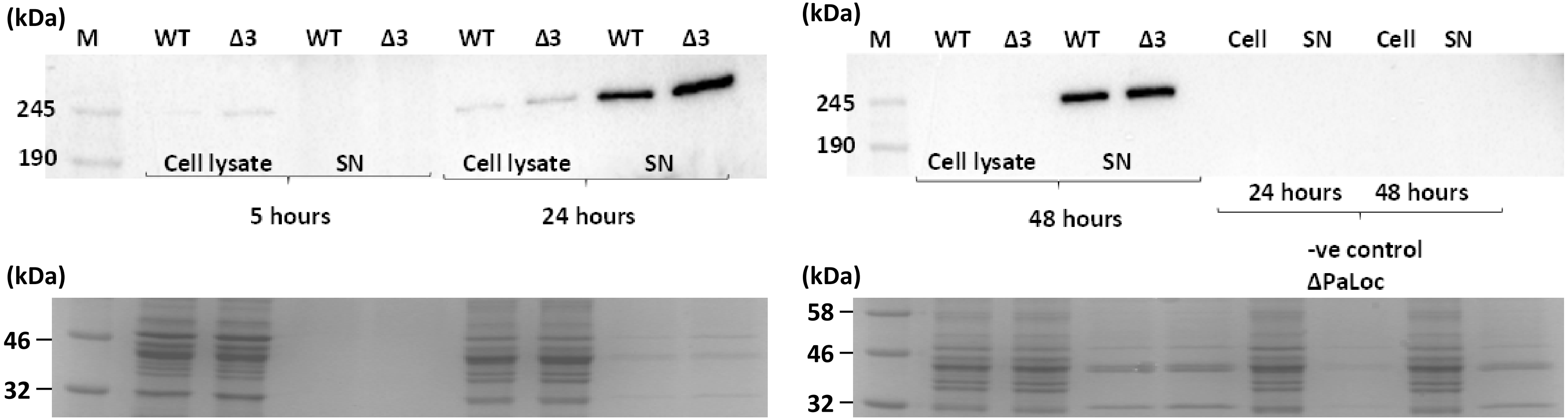

Figure S8. **Analysis of toxin release by *C. difficile* R20291 and the Δ3*ldt* mutant.** Cells were grown overnight and inoculated in TY broth at a 1/100 dilution. Cultures were harvested at different time points to prepare cell lysates and concentrated culture supernatants (SN). Toxin B was detected by Western blot (chemiluminescence) using a mouse monoclonal Anti toxin B antibody (MA1-7413) and a secondary rabbit anti-mouse antibody conjugated to horseradish peroxidase. A strain with a deletion in the gene encoding toxin B (ΔPaLoc) was used as a negative control. M, Molecular weight marker.
