# Supplementary material for: Clostridioides difficile canonical L,D-transpeptidases catalyze a novel type of peptidoglycan cross-links and are not required for beta-lactam resistance
Source: J Biol Chem. 2023 Dec 1;300(1):105529. doi: 10.1016/j.jbc.2023.105529 (PMC10792238; doi:10.1016/j.jbc.2023.105529)
Supplement: Supplementary Tables [file mmc4.pdf]

Table S1. Chemical shift assignments for the dimer peptidoglycan fragment **a.**

^{1}H signal	Chemical shift (ppm)
GlcNAc amide	8.35
GlcNAc methyl	2.08
GlcNAc anomeric	4.67
GlcNac ring	3.74, 3.58, 3.48, 3.44
MurNAc amide	8.11
MurNac methyl	2.01
MurNAc ring *	4.43, 4.32
Lac CH	4.28
Lac methyl	1.42
A NH	8.41
A CαH	4.32
A methyl	1.43
iE NH	7.92
iE CαH	4.20
iE $C\beta H_2$	1.90, 2.10
iE $C\gamma H_2$	2.26
DAP NaH **	8.11
DAP NbH **	8.04
DAP CaH **	4.43
DAP CbH **	4.26
DAP CaH_2 **	1.74
DAP CbH_2 **	1.95
DAP CcH_2 **	1.69

* Note that the muramic acid sugar has been reduced, meaning that there is no anomeric proton for this sugar.
** See structures for the locations of these protons

Table S2. Minimum inhibitory concentration (MIC) of beta lactams against *C. difficile* R20291 and ldt_{Cd} isogenic mutants.

	**WT**	**Δldt_{Cd1}**	**Δldt_{Cd2}**	**Δldt_{Cd3}**	**Δldt_{Cd1} Δldt_{Cd2}**	**Δldt_{Cd1} Δldt_{Cd3}**	**Δldt_{Cd2} Δldt_{Cd3}**	**Δldt_{Cd1} Δldt_{Cd2} Δldt_{Cd3}**
**Penicillins**								
Amoxicillin	4	4	4	4	4	4	2	2
Co-amoxicillin	2	2	1	1	2	2	2	2
Piperacillin	16	16	16	16	16	16	16	16
Piperacillin-tazobactam	16	16	16	16	16	16	16	16
**Cephalosporins**								
Cephalotin [a]	>256	>256	>256	>256	>256	>256	256	256
Cephalexin [a]	>256	>256	>256	>256	>256	>256	>256	>256
Cefuroxime [b]	>256	>256	>256	>256	>256	>256	>256	>256
Cefoxitin [b]	>256	>256	>256	>256	>256	>256	>256	>256
Ceftriaxone [c]	128	128	128	128	128	128	128	128
Cefotaxime [c]	>256	>256	>256	>256	>256	>256	>256	>256
**Carbapenems**								
Meropenem	8	8	8	8	8	8	8	8
Imipenem	32	32	16	32	16	16	16	16
**Glycopeptides**								
Vancomycin	1	1	1	1	1	1	1	2

[a] first generation cephalosporins
[b] second generation cephalosporins
[c] third generation cephalosporins

Table S3. Quantification of Ldt_{Cd2} endopeptidase activity against a 3-4 crosslinked dimer [a]

	3-4 dimer	3-4 dimer + Ldt2
**gm-AEJA=gm-AEJA**	99.963%	0.093%
**gm-AEJ=gm-AEJA - H_2O**	0	86.014%
**gm-AEJ=gm-AEJA**	0	13.861%
**gm-AEJ**	0	0.032%[b]
**gm-AEJA**	0.037%	0
	100.0%	100.0%

[a] Abundance was calculated based on Ion intensity.

[b] The low abundance of gm-AEJ corresponds to carboxy-peptidase activity on traces of gm-AEJA present in the substrate

Table S4. Bacterial strains, plasmids, and oligonucleotides.

Strains/plasmids/ oligonucleotides	Relevant properties/sequence	Source
**Strains**		
Clostridioides difficile		
R20291	Clinical isolate, ribotype 027	(1)
R20291Δ*ldt1*	R20291 derivative with an in-frame deletion in ldt_{Cd1}	This work
R20291Δ*ldt2*	R20291 derivative with an in-frame deletion in ldt_{Cd2}	This work
R20291Δ*ldt3*	R20291 derivative with an in-frame deletion in ldt_{Cd3}	This work
R20291Δ*ldt2*Δ*ldt1*	R20291 derivative with an in-frame deletion in ldt_{Cd2} *and* ldt_{Cd1}	This work
R20291Δ*ldt3*Δ*ldt1*	R20291 derivative with an in-frame deletion in ldt_{Cd3} *and* ldt_{Cd1}	This work
R20291Δ*ldt3*Δ*ldt2*	R20291 derivative with an in-frame deletion in ldt_{Cd3} *and* ldt_{Cd2}	This work
R20291Δ*ldt2*Δ*ldt1*Δ*ldt3*	R20291 derivative with an in-frame deletion in ldt_{Cd2}, ldt_{Cd1} *and* ldt_{Cd3}	This work
R20291ΔPaloc	R20291 derivative with a deletion in the genes encoding toxins	(2)
Escherichia coli		
NEB5alpha	Cloning strain	NEB
BL21(DE3)	Expression strain	NEB
CA434	HB101 derivative carrying R702	(3)
**Plasmids**		
pJAK112	Plasmid for gene replacement in *C. difficile*	(4)
pNG007	pJAK derivative containing homology regions flanking a 1332bp ldt_{Cd1}	This work
pNG008	pJAK derivative containing homology regions flanking a 1695bp ldt_{Cd2}	This work
pNG009	pJAK derivative containing homology regions flanking a 753bp ldt_{Cd3}	This work
pET2818	pET derivative for expression of His-tagged recombinant proteins	(5)
pET-Ldt1	pET2818 derivative expressing Ldt_{Cd1} (residues 3-289; C-terminal His-tag)	This work
pET-Ldt2	pET2818 derivative expressing Ldt_{Cd1} (residues 41-164; N-terminal His-tag)	This work
pET-Ldt3	pET2818 derivative expressing Ldt_{Cd1} (residues 38-469; N-terminal His-tag)	This work
**Oligonucleotides**		
RF_21 (pJAK112)	GGATTTCACATTTGCCGTTTTGTAAAC	
RF_22 (pJAK112)	GATCTTTTCTACGGGGTCTGAC	
RF1795 (ldt_{Cd1} locus)	GCTTTTACTTTGATACTGTCTGCTG	
RF1796 (ldt_{Cd1} locus)	ATGACAAACTTAAGGAAAGATGGCC	
RF1797 (ldt_{Cd2} locus)	GGATTCAGTTCCTGAATAACTAGGT	
RF1798 (ldt_{Cd2} locus)	GCAGTTGGTGAATCAGTAGAAAAAC	
RF1799 (ldt_{Cd3} locus)	GGAGGAGATATTAGAGACTATGAAG	
RF1800 (ldt_{Cd3} locus)	CGTGTTTATGCACATCCAACTATG	

(1) Stabler, R. A., He, M., Dawson, L., Martin, M., Valiente, E., Corton, C., Lawley, T. D., Sebaihia, M., Quail, M. A., Rose, G., Gerding, D. A., Gibert, M., Popoff, M. R., Parkhill, J., Dougan, G. and Wren, B. W. (2009) Comparative genome and phenotypic analysis of *Clostridium difficile* 027 strains provides insight into the evolution of a hypervirulent bacterium. *Genome Biology* **10** R102

(2) Buddle, J., Wright, R. C. T., Turner, C. E., Chaudhuri, R. R., Brockhurst, M. A. and Fagan, R. P. (2023) Multiple evolutionary pathways lead to vancomycin resistance in *Clostridioides difficile. BioRxiv*

(3) Woods, C., Humphreys, C. M., Mesquita Rodrigues, R., Ingle, P., Rowe, P., Henstra, A. M., Köpke, M., Simpson, S. D., Winzer, K. and Minton, N. P. (2019) A novel conjugal donor strain for improved DNA transfer into *Clostridium* spp. *Anaerobe* **59**:184-191

(4) Fuchs, M., Lamm-Schmidt, V., Sulzer, J., Ponath, F., Jenniches, L., Kirk, J. A., Fagan, R. P., Barquist, L., Vogel, J. and Faber, F. (2021) An RNA-centric global view of *Clostridioides difficile* reveals broad activity of Hfq in a clinically important gram-positive bacterium. *Proc Natl Acad Sci USA* **118**(25): e2103579118

(5) Eckert, C., Lecerf, M., Dubost, L., Arthur, M. and Mesnage, S. (2006) Functional analysis of AtlA, the major *N*-acetylglucosaminidase of *Enterococcus faecalis*. *J Bacteriol.* **188**(24):8513-8519
